# Screening North American plant extracts *in vitro* against *Trypanosoma brucei* for discovery of new antitrypanosomal drug leads

**DOI:** 10.1186/s12906-016-1122-0

**Published:** 2016-05-18

**Authors:** Surendra Jain, Melissa Jacob, Larry Walker, Babu Tekwani

**Affiliations:** National Center for Natural Products Research, Research Institute of Pharmaceutical Sciences, School of Pharmacy, University of Mississippi, University, MS 38677 USA; Department of BioMolecular Sciences, School of Pharmacy, University of Mississippi, University, MS 38677 USA

**Keywords:** *Trypanosoma brucei*, Natural products, Human African trypanosomiasis, North American plants

## Abstract

**Background:**

Human African Trypanosomiasis (HAT) is a protozoan parasitic disease caused by *Trypanosoma brucei*. The disease is endemic in regions of sub-Saharan Africa, covering 36 countries and more than 60 million people at the risk. Only few drugs are available for the treatment of HAT. Current drugs suffer from severe toxicities and require intramuscular or intravenous administrations. The situation is further aggravated due to the emergence of drug resistance. There is an urgent need of new drugs that are effective orally against both stages of HAT. Natural products offer an unmatched source for bioactive molecules with new chemotypes.

**Methods:**

The extracts prepared from 522 plants collected from various parts of the North America were screened in vitro against blood stage trypamastigote forms of *T. brucei*. Active extracts were further screened at concentrations ranging from 10 to 0.4 μg/mL. Active extracts were also investigated for toxicity in Differentiated THP1 cells at 10 μg/mL concentration. The results were computed for dose–response analysis and determination of IC50/IC90 values.

**Results:**

A significant number (150) of extracts showed >90 % inhibition of growth of trypomastigote blood forms of *T. brucei in* primary screening at 20 μg/mL concentration. The active extracts were further investigated for dose–response inhibition of *T. brucei* growth. The antitrypansomal activity of 125 plant extracts was confirmed with IC50 < 10 μg/mL. None of these active extracts showed toxicity against differentiated THP1 cells. Eight plants extracts namely, *Alnus rubra, Hoita macrostachya, Sabal minor, Syzygium aqueum, Hamamelis virginiana, Coccoloba pubescens, Rhus integrifolia* and *Nuphar luteum* were identified as highly potent antitrypanosomal extracts with IC_50_ values <1 μg/mL.

**Conclusions:**

Limited phytochemical and pharmacological reports are available for the lead plant extracts with potent antitrypanosomal activity. Follow up evaluation of these plant extracts is likely to yield new antitrypanosomal drug-leads or alternate medicines for treatment of HAT.

**Electronic supplementary material:**

The online version of this article (doi:10.1186/s12906-016-1122-0) contains supplementary material, which is available to authorized users.

## Background

Human African trypanosomiasis (HAT), also known as sleeping sickness, is caused by infection with *Trypanosoma brucei*. HAT is almost always fatal, if untreated or inadequately treated, and is a substantial cause of both mortality and morbidity in affected regions. Infection with *T. brucei* also has a substantial effect on livestock production [1]. The human disease occurs in two forms, depending on the subspecies of the trypanosome involved. *Trypanosoma brucei gambiense* causes a chronic infection that may persist for months or even years without major signs or symptoms of the disease. *Trypanosoma brucei rhodesiense* causes an acute infection [2], where signs and symptoms of the disease are observed a few weeks after the infective bite. The acute form develops rapidly, soon invading the central nervous system [3]. Currently, only four drugs are registered for the treatment of the HAT, which were developed several years ago. All drugs are toxic and require cumbersome treatment schedules. Pentamidine is used to treat the first stage of *T. brucei gambiense* infection [4]. Suramin is used to treat the first stage of *T. brucei rhodesiense* infection [4]. Intravenous melarsoprol is used in the second stage of both forms of the disease [5]. On average, 5 % of patients treated with melarsoprol have a fatal serious adverse event [6]. Eflornithine is used in the second stage of *T. b. gambiense* infection. No new chemical agents have been approved since eflornithine in 1990. A new protocol for the treatment of late-stage *T. brucei gambiense* that uses the combination nifurtomox/eflornithine (NECT) was recently shown to have better safety and efficacy than eflornithine alone, while being easier to administer [7]. This breakthrough represents the only new therapy for HAT since the approval of eflornithine. Toxicity and suboptimal efficacy of currently available HAT drugs, the growing problem of drug resistance to pentamidine and melarsoprol [8, 9] and severely depleted antitypanosomal drug discovery pipeline necessitate the discovery of new antitrypanosomal drugs with better efficacy and safety profiles. Natural products remain an unmatched source of drugs leads with diverse and novel chemotypes. About 70 % of the currently available drugs have their origin from natural products, mainly from plants. Screening of natural products plant extracts will promise potential for discovery of new drug leads [10]. The North American plants have shown significant medicinal values. However, the plants from this region of the world have not been explored earlier for new antiparasitic and antiprotozoal drug discovery. This study presents the results on screenings of the extracts prepared from the plants collected from this region for discovery of new natural products drug-leads and alternate medicines from traditional natural products sources for treatment of human African trypanosomiasis. *Trypansoma brucei brucei* strain 427 a non-human sub specie, which has been extensively used for molecular and biochemical investigations on trypanosomes and compounds screening, was employed for the screening [11, 12].

## Methods

### Plant collection

Botanists at the Missouri Botanical Garden, St. Louis, Missouri (MOBOT) collected the plants the plants from different parts of North America, under a cooperative scientific agreement with the National Center for Natural Products Research. The information on NPID (a unique identification number), sample name, code, genus, species, family, source, common name, plant part, geographical location, collector and collector’s number are given in the supplement Table (Additional file 1: Table S1).

### Extraction procedure

Bulk plant samples are dried and frozen in the field and freeze dried at the University of Mississippi before extraction. The freeze-dried plant materials were extracted with Dionex ASE 300. Extraction was done in 95 % ethanol. Each sample was placed in a specific ASE cell and extracted at 1500 psi, at 40 °C for 3 times with 10 min static time/extraction. ASE cells were purged for 120 s. After extraction the extracts were dried and dissolved in DMSO at a concentration of 20 mg/mL.

### Culture maintenance

Blood stage forms of *Trypanosoma brucei brucei* (bloodstream form, Strain 427 obtained from Frederick S. Buckner, Department of Medicine, University of Washington, Seattle, Washington, USA) was grown in IMDM medium supplemented with 10 % fetal bovine serum. The culture was maintained at 37 °C in 5 % CO_2_ incubator. THP1 cells obtained from ATCC were grown in RPMI 1640 medium supplemented with 10 % fetal bovine serum. The culture was maintained at 37 °C in 5 % CO2 incubator.

### Antitrypanosomal assay

A 2 days old culture of *T. brucei brucei* in the exponential phase was diluted with IMDM to 5000 parasites/mL. Maximum permissible limit of DMSO in the assay was 0.5 %. The assays were set up in clear 96 well microplates. For primary screening (Single concentration of 20 μg/mL in duplicate) extract dilutions (1 mg/mL) were prepared from the stock extracts (20 mg/mL) in IMDM medium. Each well received 4 μL of diluted extract sample and 196 μL of the culture volume (total culture volume 200 μl). The plates were incubated at 37 °C in 5 % CO_2_ for 48 h. Alamar blue (10 μl) (AbD Serotec, catalog number BUF012B) was added to each well and the plates were incubated further for overnight. Standard fluorescence was measured on a Fluostar Galaxy fluorometer (BMG LabTechnologies) at 544 nm excitation, 590 nm emission. Pentamidine and α-difluoromethylornithine (DFMO) were tested as standard (Table 1). The extracts that have shown more than 90 % inhibition of *T. brucei* growth in primary screening were subjected to secondary screening for dose–response analysis. Active extracts were screened at concentrations ranging from 10 – 0.4 μg/mL. IC_50_ and IC_90_ values were computed from dose response growth inhibition curve by XLfit version 5.2.2.

**Table 1:** In vitro antitrypanosomal activity of the most active plant extracts against *Trypanosoma brucei*

NPID	Sample name	Family	Common name	Plant part	IC50 (μg/mL)	IC90 (μg/mL)
81880	*Alnus rubra*	Betulaceae	Red alder	BK	0.94 ± 0.58	1.95 ± 0.17
81890	*Boykinia major*	Saxifragaceae	Large Boykinia	RT	2.82 ± 0.44	8.31 ± 1.50
81897	*Juniperus communis*	Cupressaceae	Juniper	LF-ST	2.40 ± 0.44	6.66 ± 2.13
81908	*Chrysolepis chrysophylla*	Fagaceae	Golden chinquapin	FL	2.89 ± 0.40	7.31 ± 1.57
81925	*Rhododendron occidentale*	Ericaceae	Western azalea	LF	2.87 ± 0.43	6.88 ± 1.16
81940	*Arctostaphylos viscida*	Ericaceae	Whiteleaf manzanita, sticky manzanita	LF	2.88 ± 0.21	5.96 ± 1.09
81953	*Eriogonum umbellatum*	Polygonaceae	Buckwheat	LF-ST	2.79 ± 0.34	6.25 ± 1.94
82438	*Eriogonum fisciculatum*	Polygonaceae	California or Eastern Mojave buckwheat	LF	2.68 ± 0.44	7.73 ± 2.33
82440	*Rhus integrifolia*	Anacardiaceae	Lemonade berry	LF	2.97 ± 0.01	4.41 ± 0.29
82453	*Hoita macrostachya*	Fabaceae	Large leatherroot	LF	0.48 ± 0.00	0.62 ± 0.00
82466	*Lepechinia calycina*	Lamiaceae	Pitcher sage; woodbalm	LF	2.50 ± 0.05	5.04 ± 0.12
82467	*Ribes speciosum*	Grossulariaceae	Fuchsia-flowered gooseberry	LF-ST-FL	2.95 ± 0.10	5.90 ± 0.08
82468	*Salvia spathacea*	Lamiaceae	Pitcher or hummingbird sage	ST	1.13 ± 0.78	3.46 ± 0.34
82484	*Sabal minor*	Arecaceae	Bush palmetto	FL	1.06 ± 0.44	2.07 ± 0.96
83334	*Medinilla magnifica*	Melastomataceae	Chandelier tree	FL-FR	2.25 ± 1.16	7.89 ± 2.48
83345	*Eucalyptus citriodora*	Myrtaceae	Lemon-scented gum	LF	2.91 ± 0.21	5.82 ± 0.04
83360	*Acer rubrum*	Sapindaceae	Red maple	LF	2.88 ± 0.50	7.45 ± 0.70
84470	*Ligustrum sinense*	Oleaceae	Privet	LF-FR	2.77 ± 0.40	4.41 ± 1.46
84516	*Hamamelis virginiana*	Hamamelidaceae	Witch hazel	ST	2.54 ± 0.53	4.65 ± 3.92
84686	*Lyonia fruticosa*	Ericaceae	Coastal plain staggerbush	ST	2.54 ± 0.71	4.65 ± 3.92
84709	*Ribes montigenum*	Grossulariaceae	Gooseberry-currant	ST	1.94 ± 0.67	7.39 ± 2.74
84712	*Quercus alba*	Fagaceae	White oak	BK	1.42 ± 0.30	7.53 ± 0.44
84715	*Leea rubra*	Vitaceae	West Indian holly, red leea	ST	1.62 ± 0.32	4.50 ± 1.04
84720	*Coccoloba pubescens*	Polygonaceae	Grandleaf seagrape	ST	0.83 ± 0.04	1.91 ± 0.16
84722	*Rhus integrifolia*	Anacardiaceae	Lemonade berry	ST	0.50 ± 0.10	1.07 ± 0.33
84738	*Nuphar luteum*	Nymphaeaceae	Water lilly	FR	0.42 ± 0.02	1.32 ± 0.11
131665	Difluoromethylornithine				5.07 ± 0.27	12.37 ± 0.96
103650	Pentamidine				0.002 ± 0.001	0.003 ± 0.001

(A complete list of plants screened is presented as supplement material –Additional file 1: Table S1 and Table S2) NPID- Natural Product Identification Details (accession number); Plant parts- BK- stem bark; LF- leaves; FL- flowers; ST- stem; FR- fruit; IC_50_ and IC_90_ values are mean ± SD

### Cytotoxicity assay

The extracts were also tested for cytotoxicity against transformed human monocytic (THP1) cells. A 4 days’ old culture of THP1 cells in the experimental phase was diluted with RPMI medium to 2.5X 10^5^ cells/mL. Phorbol 12-myristate 13-acetate (PMA) was added to the culture at 25 ng/mL concentration for transformation of the cells to adherent macrophages [13]. The PMA treated THP1 cell culture was dispensed in 96 well plates with 200 μl culture (2.5X 10^5^ cells/mL) in each well and plates were incubated at 37 °C in 5 % CO_2_ incubator for overnight. Extracts were diluted in separate plates (Daughter plates) in RPMI medium. The medium in plates with THP1 cells was replaced with fresh medium. The diluted plant extracts were added to these plates. The plates were placed again in CO_2_ incubator at 37 °C, 5 % CO_2_ for 48 h. After 48 h 10 μl of alamar blue solution was added to each well and the plates were incubated further for overnight. Standard fluorescence was measured on a fluorometer at 544 nm ex, 590 nm em. Cytotoxicity screening was done for active extracts, which have shown more than 90 % inhibition in primary *T. brucei* screening. None of the *T. brucei* active plant extracts have shown more than 50 % inhibition on differentiated THP1 cells at 10 μg/mL concentration.

## Results and discussion

The primary screening for *T. brucei* was done for extracts prepared from 522 plants collected from various parts of the North America (Additional file 1: Table S1). A significantly high number (150 extracts) of extracts showed >90 % inhibition of growth and proliferation of trypomastigote forms of *T. brucei* at 20 μg/mL. Secondary screening was done for active extracts at concentrations ranging from 10 – 0.4 μg/mL (Additional file 1: Table S2) and we identified ten plants extracts with potent antitrypanosomal activity with IC_50_ values <2 μg/mL (Table 1). Antitrypanosomal activity of these plants extracts was selective as none of these were significantly active against *Leishmania donovani*, *Plasmodium falciparum* (unpublished data) and transformed THP1 human macrophage cells (Additional file 1: Table S2). Plant extracts, those having IC_50_ less than 2 μg/mL in antitrypanosomal assay were *Alnus rubra (*0.94 μg/mL), *Hoita macrostachya* (0.48 μg/mL), *Salvia spathacea* (1.13 μg/mL), *Sabal minor* (1.06 μg/mL), *Syzygium aqueum* (1.84 μg/mL), *Rubus odoratus* (1.95 μg/mL), *Ribes montigenum* (1.94 μg/mL), *Quercus alba* (1.42 μg/mL), *Leea rubra* (1.62 μg/mL), *Coccoloba pubescens* (0.83 μg/mL), *Rhus integrifolia* (0.50 μg/mL), and *Nuphar luteum* (0.42 μg/mL). Eight additional plant extracts with IC_50_ in the range of 2.0- 2.5 μg/mL in antitrypanosomal assay were *Juniperus communis* (2.40 μg/mL), *Lepechinia calycina* (2.50 μg/mL), *Salix caroliniana* (2.24 μg/mL), *Arceuthobium occidentale* (2.47 μg/mL), *Medinilla magnifica* (2.25 μg/mL), *Acer rubrum* (2.06 μg/mL), *Pinus aristata* (2.42 μg/mL), *Hypericum hypericoides* (2.28 μg/mL) also represent new antitrypanosomal leads (Additional file 1: Table S2).

The active extracts were investigated for reported phytochemical and pharmacological activities. *Alnus rubra*, the Red alder, is a deciduous broadleaf tree native to western North America. It is the largest species of alder in North America and one of the largest in the world, reaching heights of 20–35 m. Native Americans have used various plant parts of *Alnus rubra* medicinally as a purgative, an emetic, for aching bones, headaches, coughs, biliousness, stomach problems, scrofula sores, tuberculosis, asthma, and eczema, and as a general panacea [14]. Antifungal and antibiotic activities have also been reported in *Alnus rubra* [15, 16]. Diarylheptenone 1-(3′,4’-dihydroxyphenyl)-7-(4''-hydroxyphenyl)-4-hepten-3-one and 1,7-bis(P-hydroxyphenyl)-4-hepten-3-one were isolated from *Alnus rubra* bark and their structures elucidated by spectrometric techniques [17]. A few minor diarylheptanoid glycosides namely, diarylheptanoid (S)-1,7-bis-(4-hydroxyphenyl)-heptan-3-one-5-O-beta-D-xylopyranoside, and two known compounds, 1,7-bis-(3,4-dihydroxyphenyl)-heptan-3-one-5-O-beta-D-glucopyranoside and platyphylloside were also isolated from *Alnus rubra* bark [18]. *Alnus rubra* extract is a novel antitrypanosomal lead. *Hoita macrostachya* is a species of legume known by the common name Large Leather Root. It is native to California and Baja California where it can be found in moist areas of a number of habitat types. This is a hairy, glandular perennial herb producing a tall, branching stem approaching two meters in maximum height. The potent antitrypanosomal activity reported herein is the first pharmacological activity reported in this plant. No phytochemical data are available on *Hoita macrostachya*. Therefore, this also represents a novel antitrypanosomal lead. *Sabal minor* (Arecaceae), commonly known as the Dwarf Palmetto or Bush palmetto, is one of about 14 species of Sabal palmetto palms. Native to the southeastern United States, ranging from Florida north to eastern North Carolina, and west to eastern Oklahoma and eastern Texas. Although it is mainly found in the southern states, it is one of the only palms that can withstand somewhat cooler temperatures, and has been cultivated in North and South Central Pennsylvania. This is the first report regarding antitrypanosomal activity in this plant. No phytochemical data are available on *Sabal minor. Ribes montigenum* plant commonly known as mountain gooseberry, alpine prickly currant, and gooseberry currant is native to Western North America (British Columbia to California to New Mexico). No previous phytochemical or pharmacological results are reported in this plant. *Coccoloba pubescens* (Grandleaf Seagrape; syn. *C. grandifolia*, also called “Eve’s Umbrella”) is a species of Coccoloba native to coastal regions of the Caribbean, on Antigua, Barbados, Barbuda, Dominica, Hispaniola, Martinique, Montserrat, and Puerto Rico. No phytochemical or pharmacological data are reported in this plant. *Rhus integrifolia*, also known as Lemonade Berry or Lemonade Sumac is a shrub to small tree. It is native to the Transverse and Peninsular Ranges and the South Coast regions of Southern California. This extends from Santa Barbara County and the Channel Islands to San Diego County and extending into north-central Pacific coastal Baja California and its offshore islands such as Cedros Island. This is the first report on any pharmacological activity in this plant. No phytochemical data are available on *Rhus integrifolia. Nuphar lutea*, the spatterdock, also known as yellow water-lily, cow lily, or yellow pond-lily, is an aquatic plant of the family Nymphaeaceae, native to Eurasia and North America. It grows in eutrophic freshwater beds, with its roots fixed into the ground and its leaves floating on the water’s surface. Strong inhibition of NFkappaB activity was found in extracts of leaf and rhizome from *Nuphar lutea* L. SM. (Nuphar). The inhibitory action was narrowed down to a mixture of thionupharidines and/or thionuphlutidines [19]. Antileishmanial activity has been reported in partially purified alkaloid fraction (NUP) of *Nuphar lutea* and dimeric sesquiterpene thioalkaloids were identified as the major constituents of the mixture [20]. The *Nuphar lutea* was identified as the most active antitrypanosomal plant extract with IC_50_ 0.42 μg/mL Few additional plant extracts showed activity (IC_50_) in the range of 2–10 μg/mL. Antioxidant activity has been reported in extracts from *Rhus hirta* [21], a different species of *Rhus integrifolia* (*T. brucei* IC_50_- 2.97 μg/mL), *Juniperus communis* [22] (*T. brucei* IC_50_- 2.40 μg/mL) *Sanguisorba officinalis* [23] (*T. brucei* IC_50_ 3.56 μg/mL), *Syzygium malaccense* [24] (*T. brucei* IC_50_ 2.76 μg/mL) and *Syzygium aqueum* [25] (*T. brucei* IC_50_ 1.84 μg/mL). *Eucalyptus citriodora* [26] (*T. brucei* IC_50_ 3.34 μg/mL), *Acer rubrum* [27] (*T. brucei* IC_50_ 2.07 μg/mL), *Yucca glauca* [28] (*T. brucei* IC_50_ 3.56 μg/mL) plants have also been reported for anticancer activity. Antiviral activity is reported in *Chamaecrista nictitans* [29] (*T. brucei* IC_50_ 5.76 μg/mL). Antimicrobial activity is reported in *Liriodendron tulipifera* [30] (*T. brucei* IC_50_ 4.75 μg/mL) and *Caesalpinia pulcherrima* [31] (*T. brucei* IC_50_ 4.76 μg/mL). Anthelminthic activity is reported in *Quercus alba* [32] (*T. brucei* IC_50_ 1.42 μg/mL).

## Conclusions

In conclusion, the in vitro screening of 522 extracts, prepared from plants collected from different parts of North America, against blood stage form of *T. brucei* has identified several plants extracts with potent antitrypanosomal activity and no cytotoxicity against THP1 cells. The active plants extracts namely, *Alnus rubra, Hoita macrostachya, Salvia spathacea, Sabal minor, Ribes montigenum, Quercus alba, Leea rubra, Coccoloba pubescens, Rhus integrifolia* and *Nuphar luteum* represent new antitrypanosomal leads. Most of the leadplant extracts have very limited phytochemical and pharmacological data available. Further follow up studies with these extracts are likely to provide novel compounds as potential antitrypanosomal drug leads.

### Ethics approval and consent to participate

Not applicable.

### Consent for publication

Not Applicable.

### Availability of data and materials

All the data have been included in the supplementary data. Additional data, if required, can be made available on request. The plant material listed in manuscript can be shared, if available, on a mutually agreed material transfer agreements.

## Additional file

Additional file 1: Supplemental data - Table S1 and Table S2. (PDF 370 kb)

## Abbreviations

DFMO: difluoromethyl ornithine
HAT: human african trypanosomiasis
IC50: concentration of extract producing 50 % inhibition in growth compared to controls
IC90: concentration of extract producing 90 % inhibition in growth compared to controls
MOBOT: Missouri Botanical Garden
NECT: nifurtomox/eflornithine combination
NPID: natural product identification details
PMA: phorbol 12-myristate 13-acetate
THP1: human acute monocytic leukemia cells

## References

[1] World Health O (2013). Control and surveillance of human African trypanosomiasis. World Health Organ Tech Rep Ser.

[2] Brun R, Blum J, Chappuis F, Burri C (2010). Human African trypanosomiasis. Lancet.

[3] Kennedy PG (2013). Clinical features, diagnosis, and treatment of human African trypanosomiasis (sleeping sickness). Lancet Neurol.

[4] Burri C (2010). Chemotherapy against human African trypanosomiasis: is there a road to success?. Parasitology.

[5] Lutje V, Seixas J, Kennedy A (2013). Chemotherapy for second-stage human African trypanosomiasis. Cochrane Database Syst Rev.

[6] Control and surveillance of African trypanosomiasis (1998). Report of a WHO Expert Committee. World Health Organ Tech Rep Ser.

[7] Priotto G, Kasparian S, Mutombo W, Ngouama D, Ghorashian S, Arnold U, Ghabri S, Baudin E, Buard V, Kazadi-Kyanza S (2009). Nifurtimox-eflornithine combination therapy for second-stage African Trypanosoma brucei gambiense trypanosomiasis: a multicentre, randomised, phase III, non-inferiority trial. Lancet.

[8] Gehrig S, Efferth T (2008). Development of drug resistance in Trypanosoma brucei rhodesiense and Trypanosoma brucei gambiense. Treatment of human African trypanosomiasis with natural products (Review). Int J Mol Med.

[9] Baker N, de Koning HP, Maser P, Horn D (2013). Drug resistance in African trypanosomiasis: the melarsoprol and pentamidine story. Trends Parasitol.

[10] Rollinger JM, Langer T, Stuppner H (2006). Strategies for efficient lead structure discovery from natural products. Curr Med Chem.

[11] Sykes ML, Baell JB, Kaiser M, Chatelain E, Moawad SR, Ganame D, Ioset JR, Avery VM (2012). Identification of compounds with anti-proliferative activity against Trypanosoma brucei brucei strain 427 by a whole cell viability based HTS campaign. PLoS Negl Trop Dis.

[12] Cross GA, Kim HS, Wickstead B (2014). Capturing the variant surface glycoprotein repertoire (the VSGnome) of Trypanosoma brucei Lister 427. Mol Biochem Parasitol.

[13] Jain SK, Sahu R, Walker LA, Tekwani BL. A parasite rescue and transformation assay for antileishmanial screening against intracellular Leishmania donovani amastigotes in THP1 human acute monocytic leukemia cell line. J Vis Exp. 2012;70. 10.3791/4054 PMC3577863 23299097

[14] Moerman DE (1991). The medicinal flora of Native North America: an analysis. J Ethnopharmacol.

[15] McCutcheon AR, Ellis SM, Hancock RE, Towers GH (1992). Antibiotic screening of medicinal plants of the British Columbian native peoples. J Ethnopharmacol.

[16] McCutcheon AR, Ellis SM, Hancock RE, Towers GH (1994). Antifungal screening of medicinal plants of British Columbian native peoples. J Ethnopharmacol.

[17] Chen J, Karchesy JJ, Gonzalez-Laredo RF (1998). Phenolic diarylheptenones from Alnus rubra bark. Planta Med.

[18] Chen J, Gonzalez-Laredo RF, Karchesy JJ (2000). Minor diarylheptanoid glycosides of Alnus rubra bark. Phytochemistry.

[19] Ozer J, Eisner N, Ostrozhenkova E, Bacher A, Eisenreich W, Benharroch D, Golan-Goldhirsh A, Gopas J (2009). Nuphar lutea thioalkaloids inhibit the nuclear factor kappaB pathway, potentiate apoptosis and are synergistic with cisplatin and etoposide. Cancer Biol Ther.

[20] Ozer L, El-On J, Golan-Goldhirsh A, Gopas J (2010). Leishmania major: anti-leishmanial activity of Nuphar lutea extract mediated by the activation of transcription factor NF-kappaB. Exp Parasitol.

[21] Wu T, McCallum JL, Wang S, Liu R, Zhu H, Tsao R (2013). Evaluation of antioxidant activities and chemical characterisation of staghorn sumac fruit (Rhus hirta L.). Food Chem.

[22] Yesilbag D, Cengiz SS, Cetin I, Meral Y, Biricik H (2014). Influence of juniper (Juniperus communis) oil on growth performance and meat quality as a natural antioxidant in quail diets. Br Poult Sci.

[23] Zhang S, Liu X, Zhang ZL, He L, Wang Z, Wang GS (2012). Isolation and identification of the phenolic compounds from the roots of Sanguisorba officinalis L. and their antioxidant activities. Molecules.

[24] Savitha RC, Padmavathy S, Sundhararajan A (2011). Invitro antioxidant activities on leaf extracts of syzygium malaccense (L.) merr and perry. Anc Sci Life.

[25] Osman H, Rahim AA, Isa NM, Bakhir NM (2009). Antioxidant activity and phenolic content of Paederia foetida and Syzygium aqueum. Molecules.

[26] Shen KH, Chen ZT, Duh PD (2012). Cytotoxic effect of eucalyptus citriodora resin on human hepatoma HepG2 cells. Am J Chin Med.

[27] Gonzalez-Sarrias A, Li L, Seeram NP (2012). Effects of maple (Acer) plant part extracts on proliferation, apoptosis and cell cycle arrest of human tumorigenic and non-tumorigenic colon cells. Phytother Res.

[28] Ali MS, Sharma GC, Asplund RO, Nevins MP, Garb S (1978). Isolation of antitumor polysaccharide fractions from Yucca glauca Nutt. (Lilliaceae). Growth.

[29] Herrero Uribe L, Chaves Olarte E, Tamayo Castillo G (2004). In vitro antiviral activity of Chamaecrista nictitans (Fabaceae) against herpes simplex virus: biological characterization of mechanisms of action. Rev Biol Trop.

[30] Hufford CD, Funderburk MJ, Morgan JM, Robertson LW (1975). Two antimicrobial alkaloids from heartwood of Liriodendron tulipifera L. J Pharm Sci.

[31] Sudhakar M, Rao Ch V, Rao PM, Raju DB, Venkateswarlu Y (2006). Antimicrobial activity of Caesalpinia pulcherrima, Euphorbia hirta and Asystasia gangeticum. Fitoterapia.

[32] Katiki LM, Ferreira JF, Gonzalez JM, Zajac AM, Lindsay DS, Chagas AC, Amarante AF (2013). Anthelmintic effect of plant extracts containing condensed and hydrolyzable tannins on Caenorhabditis elegans, and their antioxidant capacity. Vet Parasitol.

