# Supplementary material for: Screening North American plant extracts in vitro against Trypanosoma brucei for discovery of new antitrypanosomal drug leads
Source: BMC Complement Altern Med. 2016 May 18;16:131. doi: 10.1186/s12906-016-1122-0 (PMC4870785; doi:10.1186/s12906-016-1122-0)
Supplement: Additional file 1: — Supplemental data - Table S1 and Table S2. (PDF 370 kb) [file 12906_2016_1122_MOESM1_ESM.pdf]

Screening North American plant extracts in vitro against *Trypanosoma brucei*, the causative agent for Human African Trypanosomiasis

Surendra K. Jain[a,b], Melissa R. Jacob[a], Larry A. Walker[a,b] and Babu L. Tekwani[a,b,*]

[a]*National center for Natural products Research, Research Institute of Pharmaceutical Sciences, School of Pharmacy, University of Mississippi, University, MS, 38677, USA*

[b]*Department of BioMolecular Sciences, University of Mississippi, University, MS, 38677, USA*

btekwani@olemiss.edu

Supplement Data

Table S 1 North American plants screened in vitro against *Trypanosoma breuei, the causative aganet for human African trypanosomasis*

NPID	SampleName	Genus	Species	Family	SourceCommonName	Plant Part	Geographical Location	Collector	Collector #	% inhibition of T. brucei	Cocentation (µg/mL)	Remark
81872	*Myriophyllum hippuroides*	Myriophyllum	hippuroides	Haloragaceae	Western watermilfoil	PX	OR	Tracy Scott	75	9.40	20	
81873	*Angelica lucida*	Angelica	lucida	Apiaceae	Seacoast angelica, sea-watch	LF	OR	Tracy Scott	76	7.05	20	
81874	*Angelica lucida*	Angelica	lucida	Apiaceae	Seacoast angelica, sea-watch	ST	OR	Tracy Scott	76	1.12	20	
81875	*Angelica lucida*	Angelica	lucida	Apiaceae	Seacoast angelica, sea-watch	FR	OR	Tracy Scott	76	64.48	20	
81876	*Allium cernuum*	Allium	cernuum	Liliaceae	Nodding onion	P	CO	Greg Gust	817	4.88	20	
81877	*Ulex europaea*	Ulex	europaeus	Fabaceae	Gorse	BK	OR	Andrew Keith Townesmith	584	98.27	20	RT
81878	*Heracleum lanatum*	Heracleum	lanatum	Apiaceae	Hogweed	FL	OR	Andrew Keith Townesmith	529	27.28	20	
81879	*Digitalis purpurea*	Digitalis	purpurea	Plantaginaceae	Foxglove	FR	OR	Tracy Scott	7	19.88	20	
81880	*Alnus rubra*	Alnus	rubra	Betulaceae	Red alder	BK	OR	Tracy Scott	19	90.66	20	RT
81881	*Rumex obtusifolius*	Rumex	obtusifolius	Polygonaceae	Round-leaved dock	LF	OR	Andrew Keith Townesmith	585	NA	20	
81882	*Rumex obtusifolius*	Rumex	obtusifolius	Polygonaceae	Round-leaved dock	ST	OR	Andrew Keith Townesmith	585	0.66	20	
81883	*Rumex obtusifolius*	Rumex	obtusifolius	Polygonaceae	Round-leaved dock	FR	OR	Andrew Keith Townesmith	585	93.86	20	RT
81884	*Oxalis trilliifolia*	Oxalis	trilliifolia	Oxalidaceae	Threeleaf woodsorrel	P	OR	Andrew Keith Townesmith	587	4.01	20	
81885	*Petasites frigidus*	Petasites	frigidus	Asteraceae	Sweet coltsfoot	RT	OR	Tracy Scott	77	4.40	20	
81886	*Arctostaphylos canescens*	Arctostaphylos	canescens	Ericaceae	Hoary manzanita	LF	OR	Tracy Scott	79	97.33	20	RT
81887	*Arctostaphylos canescens*	Arctostaphylos	canescens	Ericaceae	Hoary manzanita	ST	OR	Tracy Scott	79	13.89	20	
81888	*Ceanothus integerrimus*	Ceanothus	integerrimus	Rhamnaceae	Deer brush	ST	OR	Tracy Scott	80	18.73	20	
81889	*Boykinia major*	Boykinia	major	Saxifragaceae	Large Boykinia	LF	OR	Tracy Scott	81	63.36	20	
81890	*Boykinia major*	Boykinia	major	Saxifragaceae	Large Boykinia	RT	OR	Tracy Scott	81	92.90	20	RT
81891	*Dicentra formosa*	Dicentra	formosa	Fumariaceae	Western bleeding heart, Pacific bleeding heart	P	OR	Tracy Scott	82	7.08	20	
81892	*Phacelia hastata*	Phacelia	hastata	Hydrophyllaceae	Silverleaf phacelia, scorpionweed	PX	OR	Andrew Keith Townesmith	588	94.27	20	RT
81893	*Phacelia hastata*	Phacelia	hastata	Hydrophyllaceae	Silverleaf phacelia, scorpionweed	RT	OR	Andrew Keith Townesmith	588	72.23	20	
81894	*Pseudotsuga menziesii*	Pseudotsuga	menziesii	Pinaceae	Douglas fir	FR	OR	Andrew Keith Townesmith	589	21.07	20	
81895	*Abies procera*	Abies	procera	Pinaceae	Noble fir	FR	OR	Andrew Keith Townesmith	590	99.36	20	RT
81896	*Abies procera*	Abies	procera	Pinaceae	Noble fir	BK	OR	Andrew Keith Townesmith	590	98.85	20	RT
81897	*Juniperus communis*	Juniperus	communis	Cupressaceae	Juniper	LF-ST	OR	Andrew Keith Townesmith	592	98.61	20	RT
81898	*Sorbus cf. stichensis*	Sorbus	cf. sitchensis	Rosaceae	Sitka mountain ash	FR	OR	Andrew Keith Townesmith	593	0.63	20	
81899	*Boykinia occidentalis*	Boykinia	occidentalis	Saxifragaceae	Coastal brookfoam	P	OR	Andrew Keith Townesmith	595	95.11	20	RT
81900	*Gaultheria ovatifolia*	Gaultheria	ovatifolia	Ericaceae	Mountain checkerberry	P	OR	Andrew Keith Townesmith	597	93.33	20	RT
81901	*Lupinus albifrons*	Lupinus	albifrons	Fabaceae	Silver lupine, white-leaf bush lupine, evergreen lupine	LF	OR	Andrew Keith Townesmith	598	26.87	20	
81902	*Lupinus albifrons*	Lupinus	albifrons	Fabaceae	Silver lupine, white-leaf bush lupine, evergreen lupine	ST-RT	OR	Andrew Keith Townesmith	598	12.39	20	
81903	*Lycopodium clavatum*	Lycopodium	clavatum	Lycopodiaceae	Common club moss	PX	OR	Andrew Keith Townesmith	599	14.69	20	
81904	*Ribes cereum*	Ribes	cereum	Grossulariaceae	Wax currant	ST	OR	Tracy Scott	85	NA	20	
81905	*Lotus pinnatus*	Lotus	pinnatus	Fabaceae	Meadow bird's-foot trefoil, bog bird's-foot trefoil	PX	OR	Tracy Scott	87	NA	20	
81906	*Stachys rigida*	Stachys	rigida	Lamiaceae	Rough hedgenettle	PX	OR	Tracy Scott	88	3.22	20	
81907	*Ribes lacustre*	Ribes	lacustre	Grossulariaceae	Prickly blackcurrant	PX	OR	Tracy Scott	91	91.82	20	RT
81908	*Chrysolepis chrysophylla*	Chrysolepis	chrysophylla	Fagaceae	Golden chinquapin	FL	OR	Andrew Keith Townesmith	567	97.49	20	RT
81909	*Quercus kelloggii*	Quercus	kelloggii	Fagaceae	Californian black oak	LF	OR	Andrew Keith Townesmith	601	89.18	20	
81910	*Lithocarpus densiflorus*	Lithocarpus	densiflorus	Fagaceae	Tanbark oak	ST	OR	Andrew Keith Townesmith	602	13.81	20	
81911	*Eremocarpus setigerus*	Eremocarpus	setigerus	Euphorbiaceae	Doveweed	P	OR	Andrew Keith Townesmith	603	83.54	20	
81912	*Quercus chrysolepis*	Quercus	chrysolepis	Fagaceae	Live oak	LF	OR	Andrew Keith Townesmith	604	96.33	20	RT
81913	*Hypericum calycinum*	Hypericum	calycinum	Hypericaceae	Rose of Sharon	LF	OR	Andrew Keith Townesmith	605	12.45	20	
81914	*Hypericum calycinum*	Hypericum	calycinum	Hypericaceae	Rose of Sharon	RT	OR	Andrew Keith Townesmith	605	94.05	20	RT
81915	*Prunus domestica*	Prunus	domestica	Rosaceae	Plum	BK	OR	Andrew Keith Townesmith	606	8.23	20	
81916	*Oenanthe sarmentosa*	Oenanthe	sarmentosa	Apiaceae	Water dropwort	P	OR	Andrew Keith Townesmith	607	10.60	20	
81917	*Calocedrus decurrens*	Calocedrus	decurrens	Cupressaceae	Incense cedar	BK	OR	Andrew Keith Townesmith	600	49.08	20	
81918	*Quercus chrysolepis*	Quercus	chrysolepis	Fagaceae	Live oak	BK	OR	Andrew Keith Townesmith	604	96.16	20	RT
81919	*Ribes roezlii*	Ribes	roezlii	Grossulariaceae	Sierra gooseberry	PX	OR	Andrew Keith Townesmith	608	98.61	20	RT
81920	*Vitis californica*	Vitis	californica	Vitaceae	California grape	LF	OR	Andrew Keith Townesmith	609	NA	20	
81921	*Vitis californica*	Vitis	californica	Vitaceae	California grape	ST	OR	Andrew Keith Townesmith	609	1.17	20	
81922	*Vitis californica*	Vitis	californica	Vitaceae	California grape	FR	OR	Andrew Keith Townesmith	609	1.84	20	
81923	*Mentzelia laevicaulis*	Mentzelia	laevicaulis	Loasaceae	Giiant blazing star, smoothstem blazing star	RT	OR	Tracy Scott	92	7.52	20	

81924	*Mentzelia laevicaulis*	Mentzelia	laevicaulis	Loasaceae	Giiant blazing star, smoothstem blazing star	LF	OR	Tracy Scott	92	7.03	20	
81925	*Rhododendron occidentale*	Rhododendron	occidentale	Ericaceae	Western azalea	LF	OR	Tracy Scott	93	97.16	20	RT
81926	*Rhododendron occidentale*	Rhododendron	occidentale	Ericaceae	Western azalea	ST	OR	Tracy Scott	93	17.02	20	
81927	*Aralia californica*	Aralia	californica	Araliaceae	Elk clover, California Aralia, California Spikenard	FR	OR	Tracy Scott	94	101.69	20	RT
81928	*Aralia californica*	Aralia	californica	Araliaceae	Elk clover, California Aralia, California Spikenard	ST	OR	Tracy Scott	94	NA	20	
81929	*Aralia californica*	Aralia	californica	Araliaceae	Elk clover, California Aralia, California Spikenard	LF	OR	Tracy Scott	94	100.08	20	RT
81930	*Eriodictyon californicum*	Eriodictyon	californicum	Hydrophyllaceae	Yerba santa	RT	OR	Tracy Scott	95	89.75	20	
81931	*Eriodictyon californicum*	Eriodictyon	californicum	Hydrophyllaceae	Yerba santa	LF	OR	Tracy Scott	95	21.01	20	
81932	*Darmera peltata*	Darmera	peltata	Saxifragaceae	Umbrella plant	LF	OR	Tracy Scott	96	98.03	20	RT
81933	*Darmera peltata*	Darmera	peltata	Saxifragaceae	Umbrella plant	ST	OR	Tracy Scott	96	12.12	20	
81934	*Garrya fremontii*	Garrya	fremontii	Garryaceae	Fever bush	LF	OR	Andrew Keith Townesmith	611	12.57	20	
81935	*Garrya fremontii*	Garrya	fremontii	Garryaceae	Fever bush	ST	OR	Andrew Keith Townesmith	611	20.72	20	
81936	*Amelanchier utahensis*	Amelanchier	utahensis	Rosaceae	Utah serviceberry	LF	OR	Andrew Keith Townesmith	612	0.71	20	
81937	*Amelanchier utahensis*	Amelanchier	utahensis	Rosaceae	Utah serviceberry	ST	OR	Andrew Keith Townesmith	612	3.60	20	
81938	*Cercocarpus betuloides*	Cercocarpus	betuloides	Rosaceae	Mountain mahogany	LF-FR	OR	Andrew Keith Townesmith	613	27.67	20	
81939	*Cercocarpus betuloides*	Cercocarpus	betuloides	Rosaceae	Mountain mahogany	ST	OR	Andrew Keith Townesmith	613	12.27	20	
81940	*Arctostaphylos viscida*	Arctostaphylos	viscida	Ericaceae	Whiteleaf manzanita, sticky manzanita	LF	OR	Andrew Keith Townesmith	614	99.07	20	RT
81941	*Arctostaphylos viscida*	Arctostaphylos	viscida	Ericaceae	Whiteleaf manzanita, sticky manzanita	ST	OR	Andrew Keith Townesmith	614	29.16	20	
81942	*Arctostaphylos viscida*	Arctostaphylos	viscida	Ericaceae	Whiteleaf manzanita, sticky manzanita	FR	OR	Andrew Keith Townesmith	614	12.49	20	
81943	*Sanguisorba officinalis*	Sanguisorba	officinalis	Rosaceae	Great burnet	LF	OR	Tracy Scott	98	97.34	20	RT
81944	*Sanguisorba officinalis*	Sanguisorba	officinalis	Rosaceae	Great burnet	ST-FL	OR	Tracy Scott	98	96.20	20	RT
81945	*Perideridia oregana*	Perideridia	oregana	Apiaceae	Squaw potato	PX	OR	Tracy Scott	99	NA	20	
81946	*Lithocarpus densiflorus*	Lithocarpus	densiflorus	Fagaceae	Tanbark oak	FR	OR	Andrew Keith Townesmith	602	97.51	20	RT
81947	*Pinus lambertiana*	Pinus	lambertiana	Pinaceae	Sugar pine	LF	OR	Tracy Scott	100	99.14	20	RT
81948	*Pinus lambertiana*	Pinus	lambertiana	Pinaceae	Sugar pine	ST	OR	Tracy Scott	100	11.62	20	
81949	*Pinus lambertiana*	Pinus	lambertiana	Pinaceae	Sugar pine	BK	OR	Tracy Scott	100	20.72	20	
81950	*Lonicera hispidula*	Lonicera	hispidula	Caprifoliaceae	Pink honeysuckle, California honeysuckle	P	OR	Tracy Scott	101	13.11	20	
81951	*Lonicera ciliosa*	Lonicera	ciliosa	Caprifoliaceae	Orange honeysuckle	LF-FR	OR	Tracy Scott	102	14.58	20	
81952	*Lonicera ciliosa*	Lonicera	ciliosa	Caprifoliaceae	Orange honeysuckle	ST	OR	Tracy Scott	102	0.02	20	
81953	*Eriogonum umbellatum*	Eriogonum	umbellatum	Polygonaceae	Buckwheat	LF-ST	OR	Tracy Scott	104	96.97	20	RT
81954	*Rhamnus californica*	Rhamnus	californica	Rhamnaceae	California buckthorn, coffeeberry	LF	OR	Tracy Scott	107	8.75	20	
81955	*Rhamnus californica*	Rhamnus	californica	Rhamnaceae	California buckthorn, coffeeberry	ST	OR	Tracy Scott	107	9.74	20	
81956	*Chrysothamnus nauseosus*	Chrysothamnus	nauseosus	Asteraceae	Rubber or grey rabbitbrush; chamisa	RT	OR	Tracy Scott	108	26.02	20	
81957	*Arceuthobium campylopodum*	Arceuthobium	campylopodum	Viscaceae	Western dwarf mistletoe	PX	OR	Andrew Keith Townesmith	616	92.29	20	RT
81958	*Goodyera oblongifolia*	Goodyera	oblongifolia	Orchidaceae	Western rattlesnake plantain	P	OR	Andrew Keith Townesmith	617	3.10	20	
81959	*Ceanothus cordulatus*	Ceanothus	cordulatus	Rhamnaceae	Whitethorn ceanothus, Mountain whitethorn	PX	OR	Andrew Keith Townesmith	618	95.71	20	RT
82399	*Lotus crassifolius*	Lotus	crassifolius	Fabaceae	Big deervetch; broad-leafed lotus	LF	OR	Andrew Keith Townesmith	619	3.03	20	
82400	*Lotus crassifolius*	Lotus	crassifolius	Fabaceae	Big deervetch; broad-leafed lotus	ST	OR	Andrew Keith Townesmith	619	24.49	20	
82401	*Lotus crassifolius*	Lotus	crassifolius	Fabaceae	Big deervetch; broad-leafed lotus	RT	OR	Andrew Keith Townesmith	619	89.87	20	
82402	*Lotus crassifolius*	Lotus	crassifolius	Fabaceae	Big deervetch; broad-leafed lotus	FR	OR	Andrew Keith Townesmith	619	97.76	20	RT
82403	*Rumex salicifolius*	Rumex	salicifolius	Polygonaceae	Willow dock	LF-FR	OR	Andrew Keith Townesmith	620	5.84	20	
82404	*Rumex salicifolius*	Rumex	salicifolius	Polygonaceae	Willow dock	ST	OR	Andrew Keith Townesmith	620	5.84	20	
82405	*Crataegus douglasii*	Crataegus	douglasii	Rosaceae	Black hawthorn	FR	OR	Tracy Scott	35	2.84	20	
82406	*Arbutus menziesii*	Arbutus	menziesii	Ericaceae	Madrona	FR	OR	Andrew Keith Townesmith	549	88.83	20	
82407	*Potentilla gracilis*	Potentilla	gracilis	Rosaceae	Slender Cinquefoil, or Graceful Cinquefoil	ST-FR	OR	Andrew Keith Townesmith	622	96.68	20	RT
82408	*Verbascum blattaria*	Verbascum	blattaria	Scrophulariaceae	Moth mullein	LF-RT	OR	Andrew Keith Townesmith	623	76.82	20	
82409	*Verbascum blattaria*	Verbascum	blattaria	Scrophulariaceae	Moth mullein	ST	OR	Andrew Keith Townesmith	623	11.08	20	
82410	*Verbascum blattaria*	Verbascum	blattaria	Scrophulariaceae	Moth mullein	FL	OR	Andrew Keith Townesmith	623	73.23	20	
82411	*Hylocomium cf. splendens*	Hylocomium	cf. splendens	Hylocomiaceae	Glittering wood-moss; tair-step moss; mountain fern moss	P	OR	Andrew Keith Townesmith	634	100.10	20	RT
82412	*Kindbergia oregana*	Kindbergia	oregana	Brachytheciaceae	Oregon beaked moss	P	OR	Andrew Keith Townesmith	638	43.72	20	
82413	*Quercus garryana*	Quercus	garryana	Fagaceae	Oregon white oak	ST	OR	Andrew Keith Townesmith	640	79.09	20	
82414	*Strongylodon macrobotrys*	Strongylodon	macrobotrys	Fabaceae	Jade vine	ST	MO	Andrew Keith Townesmith	644	11.83	20	
82415	*Dendrocalamus gigantea*	Dendrocalamus	gigantea	Poaceae	Giant Bamboo	ST	MO	Andrew Keith Townesmith	645	9.58	20	
82416	*Bambusa vulgaris*	Bambusa	vulgaris	Poaceae	Common bamboo	LF	MO	Andrew Keith Townesmith	646	9.99	20	
82417	*Bambusa vulgaris*	Bambusa	vulgaris	Poaceae	Common bamboo	ST	MO	Andrew Keith Townesmith	646	14.55	20	
82418	*Marantochloa leucantha*	Marantochloa	leucantha	Marantaceae	Marantochloa	LF	MO	Andrew Keith Townesmith	647	13.95	20	

82419	*Marantochloa leucantha*	Marantochloa	leucantha	Marantaceae	Marantochloa	ST	MO	Andrew Keith Townesmith	647	10.36	20	
82420	*Dioscorea bulbifera*	Dioscorea	bulbifera	Dioscoreaceae	Yam	ST	MO	Andrew Keith Townesmith	648	11.33	20	
82421	*Saccharum afficinarum*	Saccharum	officinarum	Poaceae	Sugarcane	LF	MO	Andrew Keith Townesmith	649	11.82	20	
82422	*Saccharum officinarum*	Saccharum	officinarum	Poaceae	Sugarcane	ST	MO	Andrew Keith Townesmith	649	12.39	20	
82423	*Ludovia lancifolia*	Ludovia	lancifolia	Cyclanthaceae	NULL	LF	MO	Andrew Keith Townesmith	651	99.31	20	RT
82424	*Ludovia lancifolia*	Ludovia	lancifolia	Cyclanthaceae	NULL	ST	MO	Andrew Keith Townesmith	651	7.06	20	
82425	*Solandra longifolia*	Solandra	longiflora	Solanaceae	Chalice vine	ST	MO	Andrew Keith Townesmith	652	92.99	20	RT
82426	*Clerodendron splendens*	Clerodendrum	splendens	Lamiaceae	Glorybower; bagflower; bleeding-heart	LF	MO	Andrew Keith Townesmith	653	97.93	20	RT
82427	*Clerodendron splendens*	Clerodendrum	splendens	Lamiaceae	Glorybower; bagflower; bleeding-heart	ST	MO	Andrew Keith Townesmith	653	12.57	20	
82428	*Quercus ilex*	Quercus	ilex	Fagaceae	Holly oak	LF	MO	Andrew Keith Townesmith	654	36.35	20	
82429	*Quercus ilex*	Quercus	ilex	Fagaceae	Holly oak	ST	MO	Andrew Keith Townesmith	654	98.20	20	RT
82430	*Medinilla magnifica*	Medinilla	magnifica	Melastomataceae	Chandelier tree	ST	MO	Andrew Keith Townesmith	656	98.25	20	RT
82431	*Carludovica sulcata*	Carludovica	sulcata	Cyclanthaceae	NULL	LF	MO	Andrew Keith Townesmith	663	0.49	20	
82432	*Carludovica sulcata*	Carludovica	sulcata	Cyclanthaceae	NULL	ST	MO	Andrew Keith Townesmith	663	NA	20	
82433	*Triplaris wiegeltiana*	Triplaris	weigeltiana	Polygonaceae	Longjack, Longjohn	LF	MO	Andrew Keith Townesmith	667	75.67	20	
82434	*Triplaris wiegeltiana*	Triplaris	weigeltiana	Polygonaceae	Longjack, Longjohn	ST	MO	Andrew Keith Townesmith	667	4.79	20	
82435	*Ceanothus greggi*	Ceanothus	greggii	Rhamnaceae	Desert ceanothus	LF	CA	Tracy Scott	111	95.36	20	RT
82436	*Quercus berberidifolia*	Quercus	berberidifolia	Fagaceae	California scrub oak	LF-ST	CA	Tracy Scott	112	94.61	20	RT
82437	*Garrya veatchii*	Garrya	veatchii	Garryaceae	Canyon silktassel; Veatch silktassel	ST	CA	Tracy Scott	115	NA	20	
82438	*Eriogonum fisciculatum*	Eriogonum	fasciculatum	Polygonaceae	California or Eastern Mojave buckwheat	LF	CA	Karen Meyer	4	93.94	20	RT
82439	*Heteromeles arbutifolia*	Heteromeles	arbutifolia	Rosaceae	Toyon	FR	CA	Karen Meyer	3	NA	20	
82440	*Rhus integrifolia*	Rhus	integrifolia	Anacardiaceae	Lemonade berry	LF	CA	Karen Meyer	8	99.31	20	RT
82441	*Quercus x acutidens*	Quercus	x acutidens	Fagaceae	Scrub oak	ST	CA	Andrew Keith Townesmith	674	93.94	20	RT
82442	*Phoradendron macrophyllum*	Phoradendron	macrophyllum	Viscaceae	Colorado desert mistletoe; bigleaf mistletoe; Christmas mistletoe	LF-FL	CA	Andrew Keith Townesmith	675	NA	20	
82443	*Artemisia californica*	Artemisia	californica	Asteraceae	Wormwood	ST	CA	Andrew Keith Townesmith	676	NA	20	
82444	*Baccharis salicifolia*	Baccharis	salicifolia	Asteraceae	Mule's fat, seep willow	LF-FL	CA	Andrew Keith Townesmith	685	97.64	20	RT
82445	*Ceanothus cuneatus*	Ceanothus	cuneatus	Rhamnaceae	Buckbrush	ST	CA	Andrew Keith Townesmith	692	72.04	20	
82446	*Penstemon spectablis*	Penstemon	spectabilis	Scrophulariaceae	Showy penstemon	RT	CA	Tracy Scott	134	92.03	20	RT
82447	*Vinca major*	Vinca	major	Apocynaceae	Greater periwinkle	LF	CA	Andrew Keith Townesmith	694	NA	20	
82448	*Capsella bursa-pastoris*	Capsella	bursa-pastoris	Brassicaceae	Shepherd's purse	P	CA	Andrew Keith Townesmith	699	NA	20	
82449	*Alnus rhombifolia*	Alnus	rhombifolia	Betulaceae	White alder	FL	CA	Andrew Keith Townesmith	702	94.54	20	RT
82450	*Salvia leucophylla*	Salvia	leucophylla	Lamiaceae	Purple sage	ST	CA	Tracy Scott	147	1.87	20	
82451	*Baccharis pilularis*	Baccharis	pilularis	Asteraceae	Dwarf chaparral broom	LF	CA	Tracy Scott	148	98.06	20	RT
82452	*Baccharis pilularis*	Baccharis	pilularis	Asteraceae	Dwarf chaparral broom	ST	CA	Tracy Scott	148	NA	20	
82453	*Hoita macrostachya*	Hoita	macrostachya	Fabaceae	Large leatherroot	LF	CA	Tracy Scott	151	96.77	20	RT
82454	*Fraxinus dipetala*	Fraxinus	dipetala	Oleaceae	California or two-petal ash	ST	CA	Andrew Keith Townesmith	705	NA	20	
82455	*Rhamnus californica*	Rhamnus	californica	Rhamnaceae	California buckthorn, coffeeberry	LF	CA	Andrew Keith Townesmith	708	0.93	20	
82456	*Polypodium californicum*	Polypodium	californicum	Polypodiaceae	California polypody	P	CA	Andrew Keith Townesmith	710	NA	20	
82457	*Fremontodendron californicum*	Fremontodendro n	californicum	Sterculiaceae	Flannel flower	ST	CA	Andrew Keith Townesmith	711	NA	20	
82458	*Chrysothamnus nauseousus*	Chrysothamnus	nauseosus	Asteraceae	Rubber or grey rabbitbrush; chamisa	ST	CA	Andrew Keith Townesmith	712	10.39	20	
82459	*Quercus wiszlenii*	Quercus	wislizeni	Fagaceae	Interior live oak	LF	CA	Andrew Keith Townesmith	713	63.20	20	
82460	*Ceanothus spinosa*	Ceanothus	spinosus	Rhamnaceae	Greenbark ceanothus; redheart	LF	CA	Tracy Scott	154	94.57	20	RT
82461	*Ceanothus spinosus*	Ceanothus	spinosus	Rhamnaceae	Greenbark ceanothus; redheart	ST	CA	Tracy Scott	154	93.97	20	RT
82462	*Mimulus longiflorus*	Mimulus	longiflorus	Scrophulariaceae	Southern bush Monkey-flower	ST	CA	Tracy Scott	156	NA	20	
82463	*Mimulus longiflorus*	Mimulus	longiflorus	Scrophulariaceae	Southern bush Monkey-flower	RT	CA	Tracy Scott	156	91.76	20	RT
82464	*Lotus grandiflorus*	Lotus	grandiflorus	Fabaceae	Chaparral bird's-foot trefoil	ST	CA	Karen Meyer	28	NA	20	
82465	*Malva sylvestris*	Malva	sylvestris	Malvaceae	Mallow	ST	CA	Karen Meyer	30	NA	20	
82466	*Lepechinia calycina*	Lepechinia	calycina	Lamiaceae	Pitcher sage; woodbalm	LF	CA	Karen Meyer	32	99.14	20	RT
82467	*Ribes speciosum*	Ribes	speciosum	Grossulariaceae	Fuchsia-flowered gooseberry	LF-ST-FL	CA	Andrew Keith Townesmith	715	94.44	20	RT
82468	*Salvia spathacea*	Salvia	spathacea	Lamiaceae	Pitcher or hummingbird sage	ST	CA	Tracy Scott	159	95.07	20	RT
82469	*Elymus glaucus*	Elymus	glaucus	Poaceae	Blue wild rye	FR	CA	Andrew Keith Townesmith	723	NA	20	
82470	*Verbesina cf. chapman*	Verbesina	cf. chapmanii	Asteraceae	Chapman's crownbeard	ST	FL	Andrew Keith Townesmith	730	NA	20	
82471	*Pinus palutis*	Pinus	palustris	Pinaceae	Pitch pine	ST	FL	Karen Meyer	37	NA	20	
82472	*Pteridium aquilinum*	Pteridium	aquilinum	Dennstaedtiaceae	Bracken	LF	FL	Karen Meyer	38	NA	20	
82473	*Woodwardia virginica*	Woodwardia	virginica	Blechnaceae	Virginia chainfern	LF-ST	FL	Karen Meyer	39	NA	20	
82474	*Orontium aquaticum*	Orontium	aquaticum	Araceae	Golden club	LF	FL	Karen Meyer	41	22.13	20	
82475	*Orontium aquaticum*	Orontium	aquaticum	Araceae	Golden club	FR	FL	Karen Meyer	41	4.98	20	
82476	*Leucothoe racemosa*	Leucothoë	racemosa	Ericaceae	Swamp doghobble	LF	FL	Andrew Keith Townesmith	732	8.18	20	
82477	*Hypericum crux-andreae*	Hypericum	crux-andreae	Hypericaceae	St. Peterswort	PX	FL	Karen Meyer	48	73.08	20	

82478	*Osmunda regalis*	Osmunda	regalis	Osmundaceae	Royal fern	LF-ST	FL	Karen Meyer	51	11.30	20	
82479	*Vaccinium stamineum*	Vaccinium	stamineum	Ericaceae	Deerberry	LF-ST	FL	Karen Meyer	55	NA	20	
82480	*Smilax walterii*	Smilax	walteri	Smilacaceae	Red-berried or coral greenbrier	LF-ST	FL	Andrew Keith Townesmith	743	NA	20	
82481	*Sagittaria lancifolia*	Sagittaria	lancifolia	Alismataceae	Arrow head	LF	FL	Karen Meyer	62	76.23	20	
82482	*Hydrocotyle ranunculoides*	Hydrocotyle	ranunculoides	Apiaceae	Water or floating pennywort	P	FL	Andrew Keith Townesmith	745	19.61	20	
82483	*Callicarpa americana*	Callicarpa	americana	Lamiaceae	American beautyberry	ST	FL	Andrew Keith Townesmith	746	20.27	20	
82484	*Sabal minor*	Sabal	minor	Arecaceae	Bush palmetto	FL	FL	Andrew Keith Townesmith	748	97.01	20	RT
82485	*Persea borbonia*	Persea	borbonia	Lauraceae	Red bay	LF	FL	Andrew Keith Townesmith	751	60.83	20	
82486	*Gaylussacia frondosa*	Gaylussacia	frondosa	Ericaceae	Dangleberry	ST	FL	Karen Meyer	67	12.13	20	
83278	*Osmunda regalis*	Osmunda	regalis	Osmundaceae	Royal fern	RT	FL	Karen Meyer	51	1.06	20	
83279	*Quercus inopina*	Quercus	inopina	Fagaceae	Sandhill oak	ST	FL	Andrew Keith Townesmith	756	9.76	20	
83280	*Quercus geminata*	Quercus	geminata	Fagaceae	Sand live oak	LF	FL	Andrew Keith Townesmith	757	97.05	20	RT
83281	*Pterocaulon pycnostachyum*	Pterocaulon	pycnostachyum	Asteraceae	Dense-spike blackroot	ST	FL	Karen Meyer	60	5.42	20	
83282	*Asimina reticula*	Asimina	reticulata	Annonaceae	Netted (reticulated) papaw	LF	FL	Karen Meyer	70	5.37	20	
83283	*Cladina cf. evansii*	Cladina	cf. evansii	Cladoniaceae	Reindeer Lichen	P	FL	Andrew Keith Townesmith	777	5.11	20	
83284	*Pinus serotina*	Pinus	serotina	Pinaceae	Pond pine	BK	FL	Karen Meyer	102	91.36	20	RT
83285	*Sabatia calycina*	Sabatia	calycina	Gentianaceae	Coastal rose gentian	PX	FL	Andrew Keith Townesmith	783	13.23	20	
83286	*Rumex verticillata*	Rumex	verticillatus	Polygonaceae	Swamp dock	LF	FL	Andrew Keith Townesmith	784	NA	20	
83287	*Ptilimnium capillaceum*	Ptilimnium	capillaceum	Apiaceae	Herbwilliam	P	FL	Andrew Keith Townesmith	787	0.43	20	
83288	*Vitis cinerea*	Vitis	cinerea	Vitaceae	Sweet winter grape	FL	FL	Andrew Keith Townesmith	750	NA	20	
83289	*Eriocaulon anceps*	Eriocaulon	anceps	Eriocaulaceae	Whitehead bogbutton	P	FL	Andrew Keith Townesmith	788	95.91	20	RT
83290	*Chapmannia floridana*	Chapmannia	floridana	Fabaceae	Florida alicia	P	FL	Karen Meyer	105	7.49	20	
83291	*Salix caroliniana*	Salix	caroliniana	Salicaceae	Coastal plain willow	LF	FL	Andrew Keith Townesmith	797	90.16	20	RT
83292	*Rhynchospora colorata*	Rhynchospora	colorata	Cyperaceae	Starrush whitetop	P	FL	Karen Meyer	109	9.85	20	
83293	*Polygala lutea*	Polygala	lutea	Polygalaceae	Orange milkwort	P	FL	Andrew Keith Townesmith	798	13.86	20	
83294	*Berlandiera subacaulis*	Berlandiera	subacaulis	Asteraceae	Florida greeneyes	LF	FL	Andrew Keith Townesmith	799	91.28	20	RT
83295	*Mayaca fluviatilis*	Mayaca	fluviatilis	Mayacaceae	Stream bogmoss	P	FL	Andrew Keith Townesmith	801	65.53	20	
83296	*Hydrocotyle umbellata*	Hydrocotyle	umbellata	Apiaceae	Manyflower marsh pennywort	LF-ST	FL	Andrew Keith Townesmith	802	50.81	20	
83297	*Lotus purshianus*	Lotus	purshianus	Fabaceae	Spanish lotus	PX	CA	Karen Meyer	166	3.42	20	
83298	*Cupressus lawsoniana*	Cupressus	lawsoniana	Cupressaceae	Lawson's cypress	LF	CA	Andrew Keith Townesmith	864	76.41	20	
83299	*Allium validum*	Allium	validum	Liliaceae	Swamp onion	PX	CA	Andrew Keith Townesmith	876	5.13	20	
83300	*Gentiana calycosa*	Gentiana	calycosa	Gentianaceae	Gentian	PX	CA	Andrew Keith Townesmith	878	8.43	20	
83301	*Eriophorum criniger*	Eriophorum	crinigerum	Cyperaceae	Fringed cottongrass	PX	CA	Andrew Keith Townesmith	883	11.28	20	
83302	*Darlingtonia californica*	Darlingtonia	californica	Sarraceniaceae	California pitcher plant, cobra lily, cobra plant (carnivorous plant)	LF	CA	Andrew Keith Townesmith	888	3.61	20	
83303	*Orthocarpus cuspidatus*	Orthocarpus	cuspidatus	Scrophulariaceae	Toothed owl's-clover	P	CA	Karen Meyer	187	2.31	20	
83304	*Salix lemmonii*	Salix	lemmonii	Salicaceae	Lemmon's willow	ST	CA	Karen Meyer	188	26.63	20	
83305	*Acer negundo*	Acer	negundo	Sapindaceae	Box elder	FR	CA	Andrew Keith Townesmith	892	6.00	20	
83306	*Cornus glabrata*	Cornus	glabrata	Cornaceae	Brown dogwood, western cornel	LF	CA	Andrew Keith Townesmith	893	15.01	20	
83307	*Eriogonum nudum*	Eriogonum	nudum	Polygonaceae	Naked buckwheat	ST-FL	CA	Karen Meyer	189	98.83	20	RT
83308	*Ceanothus integerrimus*	Ceanothus	integerrimus	Rhamnaceae	Deer brush	FR	CA	Andrew Keith Townesmith	853	13.93	20	
83309	*Arceuthobium occidentale*	Arceuthobium	occidentale	Viscaceae	Gray pine dwarf mistletoe	P	CA	Karen Meyer	192	97.32	20	RT
83310	*Rhamnus tomentella*	Rhamnus	tomentella	Rhamnaceae	Mountain coffeeberry	ST	CA	Karen Meyer	193	0.46	20	
83311	*Cinnamomum verum*	Cinnamomum	verum	Lauraceae	Cinnamon	LF	HI	Andrew Keith Townesmith	1017	1.64	20	
83312	*Musa velutina*	Musa	velutina	Musaceae	Pink banana	LF	HI	Andrew Keith Townesmith	1018	18.11	20	
83313	*Angiopteris evecta*	Angiopteris	evecta	Marattiaceae	Oriental vessel fern	LF	HI	Andrew Keith Townesmith	1019	2.98	20	
83314	*Impatiens walleriana*	Impatiens	walleriana	Balsaminaceae	Busy Lizzy	LF	HI	Andrew Keith Townesmith	1020	6.03	20	
83315	*Musa velutina*	Musa	velutina	Musaceae	Pink banana	FL	HI	Andrew Keith Townesmith	1018	6.24	20	
83316	*Araucaria cunninghamii*	Araucaria	cunninghamii	Araucariaceae	Hoop pine	LF	HI	Karen Meyer	303	11.54	20	
83317	*Spathiphyllum wallisii*	Spathiphyllum	wallisii	Araceae	Peace lily	LF	HI	Karen Meyer	304	101.28	20	RT
83318	*Spathiphyllum wallisii*	Spathiphyllum	wallisii	Araceae	Peace lily	FL	HI	Karen Meyer	304	-0.72	20	
83319	*Syzygium malaccense*	Syzygium	malaccense	Myrtaceae	Malay apple	LF	HI	Karen Meyer	305	98.62	20	RT
83320	*Pleiostachya pruniosa*	Pleiostachya	pruinosa	Marantaceae	Platanillo, prayer plant	LF	HI	Karen Meyer	307	NA	20	
83321	*Cecropia obtusifolia*	Cecropia	obtusifolia	Urticaceae	Trumpet tree	LF	HI	Karen Meyer	309	8.85	20	
83322	*Musa ornata*	Musa	ornata	Musaceae	Flowering or ornamental banana	FL	HI	Karen Meyer	306	4.71	20	
83323	*Elaeocarpus angustifolius*	Elaeocarpus	angustifolius	Elaeocarpaceae	Blue marble tree, blue fig, blue quandong	LF	HI	Andrew Keith Townesmith	1021	98.08	20	RT
83324	*Ficus drupacea*	Ficus	drupacea	Moraceae	Brown wooly fig	LF	HI	Andrew Keith Townesmith	1023	13.30	20	
83325	*Begonia hirtella*	Begonia	hirtella	Begoniaceae	Brazilian begonia	P	HI	Andrew Keith Townesmith	1025	16.92	20	
83326	*Cinnamomum burmannii*	Cinnamomum	burmannii	Lauraceae	Padang cassia	LF	HI	Andrew Keith Townesmith	1026	4.54	20	
83327	*Tabebuia rosea*	Tabebuia	rosea	Bignoniaceae	Roble de Sabana, trumpet tree	LF	HI	Karen Meyer	310	3.25	20	
83328	*Microsorum scalopendria*	Microsorum	scolopendria	Polypodiaceae	Embossed fern	LF	HI	Karen Meyer	311	1.44	20	
83329	*Syzygium aqueum*	Syzygium	aqueum	Myrtaceae	Water cherry, watery rose apple, Lau Lau	LF	HI	Karen Meyer	312	99.95	20	RT

83330	*Spathiphyllum cannifolium*	Spathiphyllum	cannifolium	Araceae	Peace lily	LF	HI	Karen Meyer	313	6.39	20	
83331	*Spathiphyllum cannifolium*	Spathiphyllum	cannifolium	Araceae	Peace lily	FL	HI	Karen Meyer	313	7.69	20	
83332	*Clerodendrum macrostegium*	Clerodendrum	macrostegium	Lamiaceae	Velvetleaf glorybower	LF	HI	Karen Meyer	314	88.80	20	
83334	*Medinilla magnifica*	Medinilla	magnifica	Melastomataceae	Chandelier tree	FL-FR	HI	Karen Meyer	318	97.25	20	RT
83335	*Ficus trigonata*	Ficus	trigonata	Moraceae	Jaguey blanco, wild fig	LF	HI	Karen Meyer	319	99.81	20	RT
83336	*Cordyline fruticosa*	Cordyline	fruticosa	Asparagaceae	Cabbage palm, good luck plant, palm lily, ti plant	LF	HI	Andrew Keith Townesmith	1028	2.55	20	
83337	*Ardisia crenata*	Ardisia	crenata	Myrsinaceae	Coralberry	LF	HI	Andrew Keith Townesmith	1032	77.99	20	
83338	*Christella dentata*	Christella	dentata	Thelypteridaceae	Downy wood fern	PX	HI	Karen Meyer	322	7.18	20	
83339	*Macaranga mappa*	Macaranga	mappa	Euphorbiaceae	Pengua	LF	HI	Karen Meyer	323	88.98	20	
83340	*Erythrina poeppigiana*	Erythrina	poeppigiana	Fabaceae	Mountain immortelle	LF	HI	Karen Meyer	324	13.17	20	
83341	*Costus productus*	Costus	productus	Costaceae	Productus	FL	HI	Karen Meyer	326	14.88	20	
83342	*Zingiber spectabile*	Zingiber	spectabile	Zingiberaceae	Beehive plant	FL	HI	Karen Meyer	327	NA	20	
83343	*Alpinia intermedia*	Alpinia	intermedia	Zingiberaceae	Variegated ginger	LF	HI	Karen Meyer	329	89.51	20	
83344	*Pleuranthodium hellwigii*	Pleuranthodium	hellwigii	Zingiberaceae	NULL	LF	HI	Karen Meyer	332	53.40	20	
83345	*Eucalyptus citriodora*	Eucalyptus	citriodora	Myrtaceae	Lemon-scented gum	LF	HI	Andrew Keith Townesmith	1034	98.52	20	RT
83346	*Psilotum nudum*	Psilotum	nudum	Psilotaceae	Whiskfern	PX	HI	Andrew Keith Townesmith	1036	12.53	20	
83347	*Schefflera actinophylla*	Schefflera	actinophylla	Araliaceae	Umbrella tree, octopus tree, amate	LF	HI	Andrew Keith Townesmith	1037	100.01	20	RT
83348	*Aglaonema cf. modestum*	Aglaonema	cf. modestum	Araceae	Chinese evergreen	LF	HI	Andrew Keith Townesmith	1038	9.88	20	
83349	*Roystonea cf. oleracea*	Roystonea	cf. oleracea	Arecaceae	Caribbean royal palm, imperial palm, cabbage palm	LF	HI	Andrew Keith Townesmith	1041	42.20	20	
83350	*Dillenia indica*	Dillenia	indica	Dilleniaceae	Chulta, elephant apple	LF	HI	Karen Meyer	308	NA	20	
83351	*Eucalyptus citriodora*	Eucalyptus	citriodora	Myrtaceae	Lemon-scented gum	BK	HI	Andrew Keith Townesmith	1034	96.17	20	RT
83352	*Falcataria moluccana*	Falcataria	moluccana	Fabaceae	Peacocksplume	LF	HI	Karen Meyer	342	60.39	20	
83353	*Passiflora auberosa*	Passiflora	suberosa	Passifloraceae	Corkystem passionflower	LF-ST	HI	Andrew Keith Townesmith	1048	3.33	20	
83354	*Mimosa pudica*	Mimosa	pudica	Fabaceae	Sensitive plant	P	HI	Karen Meyer	343	52.10	20	
83355	*Kyllinga memoralis*	Kyllinga	nemoralis	Cyperaceae	Whitehead spikesedge	P	HI	Karen Meyer	344	64.01	20	
83356	*Casuarina equisetifolia*	Casuarina	equisetifolia	Casuarinaceae	Agoho she-oak, filao tree	LF	HI	Andrew Keith Townesmith	1051	41.58	20	
83357	*Ardisia elliptica*	Ardisia	elliptica	Myrsinaceae	Shoebutton	LF	HI	Karen Meyer	385	101.33	20	RT
83358	*Nymphaea odorata*	Nymphaea	odorata	Nymphaeaceae	Fragrant water lily	FL	WI	Karen Meyer	861	97.70	20	RT
83359	*Echinocystis lobata*	Echinocystis	lobata	Cucurbitaceae	Wild cucumber	FR	WI	Karen Meyer	862	NA	20	
83360	*Acer rubrum*	Acer	rubrum	Sapindaceae	Red maple	LF	WI	Karen Meyer	863	93.93	20	RT
83361	*Acer rubrum*	Acer	rubrum	Sapindaceae	Red maple	ST	WI	Karen Meyer	863	93.35	20	RT
83362	*Agrimonia gryposepala*	Agrimonia	gryposepala	Rosaceae	Tall, hairy agrimony	LF-FR	WI	Karen Meyer	864	11.72	20	
83363	*Agrimonia gryposepala*	Agrimonia	gryposepala	Rosaceae	Tall, hairy agrimony	ST	WI	Karen Meyer	864	8.65	20	
83364	*Verbesina virginica*	Verbesina	virginica	Asteraceae	White crownbeard	LF-FL	MO	Karen Meyer	865	100.21	20	RT
83365	*Verbesina virginica*	Verbesina	virginica	Asteraceae	White crownbeard	ST	MO	Karen Meyer	865	29.39	20	
83938	*Chamaecrista nictitans*	Chamaecrista	nictitans	Fabaceae	Sensitive partridge pea	P	HI	Andrew Keith Townesmith	1094	90.68	20	RT
83939	*Pinus ponderosa*	Pinus	ponderosa	Pinaceae	Ponderosa pine	BK	NM	John Stone	3799	12.78	20	
83940	*Acer glabrum*	Acer	glabrum	Sapindaceae	Rock maple	ST	CO	John Stone	3826	25.96	20	
83941	*Betula fontinalis*	Betula	fontinalis	Betulaceae	Water birch	ST	CO	John Stone	3839	14.42	20	
83942	*Lithospermum incisum*	Lithospermum	incisum	Boraginaceae	Gromwell	P	CO	John Stone	3843	9.20	20	
83943	*Prunus virginiana*	Prunus	virginiana	Rosaceae	Chokecherry	ST	CO	John Stone	3844	6.33	20	
83944	*Geranium richardsonii*	Geranium	richardsonii	Geraniaceae	Richardson's geranium	P	CO	Greg Gust	163	98.36	20	RT
83945	*Heracleum sphondylium*	Heracleum	sphondylium	Apiaceae	Common hogweed	P	CO	Greg Gust	165	88.50	20	
83946	*Sedum lanceolatum*	Sedum	lanceolatum	Crassulaceae	Spearleaf stonecrop	P	CO	John Stone	3850	0.77	20	
83947	*Arnica cordifolia*	Arnica	cordifolia	Asteraceae	Heartleaf arnica	P	CO	John Stone	3851	95.13	20	RT
83948	*Collomia linearis*	Collomia	linearis	Polemoniaceae	Tiny trumpet	P	CO	John Stone	3866	4.16	20	
83949	*Leccinum insigne*	Leccinum	insigne	Boletaceae	Orange cap, scaber stalk, Aspen bolete	P	CO	John Stone	3871	NA	20	
83950	*Berberis nervosa*	Berberis	nervosa	Berberidaceae	Cascade barberry	RT	WA	Allison Colwell	122	38.56	20	
83951	*Trifolium parryi*	Trifolium	parryi	Fabaceae	Parry's clover	P	CO	John Stone	3892	19.43	20	
83952	*Pueraria lobata*	Pueraria	lobata	Fabaceae	Kudzu	LF-ST	NC	John Stone	1554	28.43	20	
83953	*Ribes inerme*	Ribes	inerme	Grossulariaceae	Whitestem gooseberry	PX	CO	Greg Gust	214	70.15	20	
83954	*Pinus aristata*	Pinus	aristata	Pinaceae	Bristle-cone pine	P	CO	John Stone	3898	99.39	20	RT
83955	*Pedicularis bracteosa*	Pedicularis	bracteosa	Orobanchaceae	Bracted lousewort	P	CO	Greg Gust	178	3.11	20	
83956	*Vaccinium scoparium*	Vaccinium	scoparium	Ericaceae	Grouseberry	P	CO	Greg Gust	179	93.32	20	RT
83957	*Arnica mollis*	Arnica	mollis	Asteraceae	Arnica	P	CO	John Stone	3899	91.41	20	RT
83958	*Limnorchis dilitata*	Limnorchis	dilatata	Orchidaceae	White bog-orchid	PX	CO	Greg Gust	184	17.81	20	
83959	*Saxifraga odontoloma*	Saxifraga	odontoloma	Saxifragaceae	Brook saxifrage	P	CO	Greg Gust	188	98.69	20	RT
83960	*Carex cf. lanufinosa*	Carex	cf. lanuginosa	Cyperaceae	Wooly sedge	P	CO	Greg Gust	194	9.74	20	
83961	*Ribes montigenum*	Ribes	montigenum	Grossulariaceae	Gooseberry-currant	PX	CO	Greg Gust	195	97.69	20	RT
83962	*Carex nebrascensis*	Carex	nebrascensis	Cyperaceae	Nebraska sedge	P	CO	Greg Gust	199	NA	20	
83963	*Rubus deliciosus*	Rubus	deliciosus	Rosaceae	Rocky Mountain raspberry	PX	CO	Greg Gust	215	96.12	20	RT
83964	*Lycopodium lucidulum*	Lycopodium	lucidulum	Lycopodiaceae	Shining club moss	P	NC	John Stone	1682	4.93	20	

83965	*Artemisia cana*	Artemisia	cana	Asteraceae	Wormwood	PX	CO	Greg Gust	203	10.38	20	
83966	*Carex microptera*	Carex	microptera	Cyperaceae	Smallwing sedge	P	CO	Greg Gust	204	6.82	20	
83967	*Phacelia heterophylla*	Phacelia	heterophylla	Hydrophyllaceae	Varileaf phacelia	P	CO	John Stone	3900	14.13	20	
83968	*Delphinium glaucum*	Delphinium	glaucum	Ranunculaceae	Giant larkspur	P	CO	John Stone	3902	8.57	20	
83969	*Aconitum columbianum*	Aconitum	columbianum	Ranunculaceae	Columbian monkshood	P	CO	John Stone	3903	35.39	20	
83970	*Arctostaphylos uva-ursi*	Arctostaphylos	uva-ursi	Ericaceae	Bearberry	LF	CO	John Stone	3904	85.19	20	
83971	*Linum lewisii*	Linum	lewisii	Linaceae	Lewis flax	P	CO	John Stone	3905	10.33	20	
83972	*Ribes cf. leptanthum*	Ribes	cf. leptanthum	Grossulariaceae	Trumpet gooseberry	PX	CO	John Stone	3907	96.34	20	RT
83973	*Castilleja rhexiifolia*	Castilleja	rhexiifolia	Scrophulariaceae	Splitleaf Indian paintbrush	P	CO	John Stone	3911	7.95	20	
83974	*Vaccinium myrtillus*	Vaccinium	myrtillus	Ericaceae	Bilberry	P	CO	John Stone	3912	82.68	20	
83975	*Zigadenus elegans*	Zigadenus	elegans	Liliaceae	Mountain death-camas	P	CO	John Stone	3914	12.36	20	
83976	*Stachys palustris*	Stachys	palustris	Lamiaceae	Marsh woundwort	P	CO	Greg Gust	208	11.05	20	
83977	*Grindelia squarrosa*	Grindelia	squarrosa	Asteraceae	Rosin weed	P	CO	Greg Gust	210	15.73	20	
83978	*Conium maculatum*	Conium	maculatum	Apiaceae	Hemlock	ST	CO	Greg Gust	211	NA	20	
83979	*Prunus virginiana*	Amelanchier	alnifolia	Rosaceae	Saskatoon	PX	CO	Greg Gust	216	6.22	20	
83980	*Berteroa incana*	Berteroa	incana	Brassicaceae	Hoary alyssum	P	CO	Greg Gust	217	NA	20	
83981	*Convolvulus arvensis*	Convolvulus	arvensis	Convolvulaceae	Field bindweed	P	CO	Greg Gust	218	98.45	20	RT
83982	*Phalaris arundinacea*	Phalaris	arundinacea	Poaceae	Canary grass	P	CO	John Stone	3916	5.38	20	
83983	*Medicago sativa*	Medicago	sativa	Fabaceae	Alfalfa	PX	CO	John Stone	3917	5.87	20	
83984	*Agropyron desetorum*	Agropyron	desertorum	Poaceae	Desert wheatgrass	P	CO	John Stone	3918	13.45	20	
83985	*Yucca glauca*	Yucca	glauca	Agavaceae	Soapweed	LF	CO	John Stone	3920	28.18	20	
83986	*Yucca glauca*	Yucca	glauca	Agavaceae	Soapweed	ST	CO	John Stone	3920	99.54	20	RT
83987	*Yucca glauca*	Yucca	glauca	Agavaceae	Soapweed	FR	CO	John Stone	3920	33.37	20	
83988	*Opuntia phaeacantha*	Opuntia	phaeacantha	Cactaceae	Bastard fig	PX	CO	John Stone	3925	1.06	20	
83989	*Elymus canadensis*	Elymus	canadensis	Poaceae	Canadian wild rye	PX	CO	John Stone	3926	2.97	20	
83990	*Cleome serrulata*	Cleome	serrulata	Capparaceae	Rocky Mountain beeplant	PX	CO	John Stone	3927	4.53	20	
83991	*Bahia oppositifolia*	Bahia	oppositifolia	Asteraceae	Oppositeleaf bahia	P	CO	John Stone	3928	99.77	20	RT
83992	*Thelesperma gracile*	Thelesperma	gracile	Asteraceae	Greenthread	P	CO	John Stone	3930	13.61	20	
83993	*Asclepias hallii*	Asclepias	hallii	Asclepiadaceae	Purple silkweed	ST	CO	John Stone	3932	13.75	20	
83994	*Asclepias hallii*	Asclepias	hallii	Asclepiadaceae	Purple silkweed	LF-FL	CO	John Stone	3932	87.98	20	
83995	*Senecio spartioides*	Senecio	spartioides	Asteraceae	Ragwort/groundsel	PX	CO	John Stone	3933	99.56	20	RT
83996	*Pyrularia pubera*	Pyrularia	pubera	Santalaceae	Oil nut	LF	NC	John Stone	3934	8.76	20	
83997	*Pyrularia pubera*	Pyrularia	pubera	Santalaceae	Oil nut	ST	NC	John Stone	3934	4.88	20	
83998	*Eupatorium rugosum*	Eupatorium	rugosum	Asteraceae	Thoroughwort	P	NC	John Stone	3935	42.14	20	
83999	*Erigeron annuus*	Erigeron	annuus	Asteraceae	Annual fleabane	P	NC	John Stone	3936	79.32	20	
84000	*Helianthus microcephalus*	Helianthus	microcephalus	Asteraceae	Small woodland sunflower	P	NC	John Stone	3937	52.78	20	
84001	*Hydrangea arborescens*	Hydrangea	arborescens	Hydrangeaceae	Seven barks	PX	NC	John Stone	3939	40.06	20	
84002	*Magnolia fraseri*	Magnolia	fraseri	Magnoliaceae	Mountain magnolia	LF	NC	John Stone	3940	3.38	20	
84003	*Magnolia fraseri*	Magnolia	fraseri	Magnoliaceae	Mountain magnolia	ST	NC	John Stone	3940	98.89	20	RT
84004	*Magnolia fraseri*	Magnolia	fraseri	Magnoliaceae	Mountain magnolia	FL	NC	John Stone	3940	38.92	20	
84005	*Asclepias exaltata*	Asclepias	exaltata	Asclepiadaceae	Poke milkweed	LF-ST	NC	John Stone	3941	9.53	20	
84006	*Asclepias exaltata*	Asclepias	exaltata	Asclepiadaceae	Poke milkweed	FR	NC	John Stone	3941	99.66	20	RT
84007	*Prunella vulgaris*	Prunella	vulgaris	Lamiaceae	Self-heal	PX	NC	John Stone	3942	11.37	20	
84008	*Rhododendron catawbiense*	Rhododendron	catawbiense	Ericaceae	Catawba rosebay	LF	NC	John Stone	3943	93.50	20	RT
84009	*Rhododendron catawbiense*	Rhododendron	catawbiense	Ericaceae	Catawba rosebay	ST	NC	John Stone	3943	95.74	20	RT
84010	*Rhododendron catawbiense*	Rhododendron	catawbiense	Ericaceae	Catawba rosebay	FR	NC	John Stone	3943	95.31	20	RT
84011	*Kalmia latifolia*	Kalmia	latifolia	Ericaceae	Mountain laurel	LF-FR	NC	John Stone	3944	36.56	20	
84012	*Gaylussacia ursina*	Gaylussacia	ursina	Ericaceae	Bear huckleberry	LF-FR	NC	John Stone	3945	1.09	20	
84013	*Ilex ambigua*	Ilex	ambigua	Aquifoliaceae	Carolina holly	LF-FR	NC	John Stone	3946	4.90	20	
84014	*Ilex ambigua*	Ilex	ambigua	Aquifoliaceae	Carolina holly	ST	NC	John Stone	3946	93.15	20	RT
84015	*Collinsonia canadensis*	Collinsonia	canadensis	Lamiaceae	Canada horsebalm, richweed, heal-all	P	NC	John Stone	3947	15.32	20	
84016	*Campanula divaricata*	Campanula	divaricata	Campanulaceae	Small bonny bellflower	P	NC	John Stone	3948	55.98	20	
84017	*Gaylussacia ursina*	Gaylussacia	ursina	Ericaceae	Bear huckleberry	ST	NC	John Stone	3945	65.14	20	
84018	*Actaea pachpoda*	Actaea	pachypoda	Ranunculaceae	White baneberry, doll's eyes	P	NC	John Stone	3949	7.90	20	
84019	*Elaeagnus umbellata*	Elaeagnus	umbellata	Elaeagnaceae	Autumn olive	FR	NC	John Stone	3951	NA	20	
84020	*Elaeagnus umbellata*	Elaeagnus	umbellata	Elaeagnaceae	Autumn olive	ST	NC	John Stone	3951	95.91	20	RT
84021	*Elaeagnus umbellata*	Elaeagnus	umbellata	Elaeagnaceae	Autumn olive	LF	NC	John Stone	3951	27.69	20	
84022	*Tsuga canadensis*	Tsuga	canadensis	Pinaceae	Canadian hemlock	PX	NC	John Stone	3952	93.42	20	RT
84023	*Acer pensylvanicum*	Acer	pensylvanicum	Sapindaceae	Moosewood, striped maple	ST	NC	John Stone	3953	98.48	20	RT
84024	*Helianthus giganteus*	Helianthus	giganteus	Asteraceae	Giant sunflower	PX	NC	John Stone	3954	97.94	20	RT
84025	*Aristolochia macrophylla*	Aristolochia	macrophylla	Aristolochiaceae	Pipevine	PX	NC	John Stone	3955	79.31	20	
84458	*Rhododendron cf. nudiflorum*	Rhododendron	cf. nudiflorum	Ericaceae	Pink azalea	PX	NC	John Stone	3956	0.45	20	
84459	*Ligusticum canadense*	Ligusticum	canadense	Apiaceae	Canadian licorice root	P	NC	John Stone	3957	0.95	20	

84460	*Thalictrum pubescens*	Thalictrum	pubescens	Ranunculaceae	Tall meadow-rue, King of the Meadow	P	NC	John Stone	3958	10.98	20	
84461	*Solidago caesia*	Solidago	caesia	Asteraceae	Wreath goldenrod	PX	NC	John Stone	3959	89.74	20	
84462	*Viburnum nudum*	Viburnum	nudum	Adoxaceae	Smooth withe rod, possumhaw	LF	NC	John Stone	3960	7.28	20	
84463	*Viburnum nudum*	Viburnum	nudum	Adoxaceae	Smooth withe rod, possumhaw	FR	NC	John Stone	3960	98.88	20	RT
84465	*Diervilla sessilifolia*	Diervilla	sessilifolia	Caprifoliaceae	Southern bush honeysuckle	ST	NC	John Stone	3961	100.02	20	RT
84466	*Vaccinium pallidum*	Vaccinium	pallidum	Ericaceae	Blue ridge blueberry, early lowbush blueberry	PX	NC	John Stone	3962	93.34	20	RT
84467	*Magnolia tripetala*	Magnolia	tripetala	Magnoliaceae	Umbrella magnolia, umbrella tree	LF	NC	Greg Gust	220	99.83	20	RT
84468	*Magnolia tripetala*	Magnolia	tripetala	Magnoliaceae	Umbrella magnolia, umbrella tree	FR	NC	Greg Gust	220	99.56	20	RT
84470	*Ligustrum sinense*	Ligustrum	sinense	Oleaceae	Privet	LF-FR	NC	Greg Gust	221	96.73	20	RT
84471	*Ligustrum sinense*	Ligustrum	sinense	Oleaceae	Privet	ST	NC	Greg Gust	221	96.50	20	RT
84472	*Leucothoe axillaris*	Leucothoe	axillaris	Ericaceae	Coastal doghobble	ST	NC	Greg Gust	223	93.57	20	RT
84473	*Leucothoe axillaris*	Leucothoe	axillaris	Ericaceae	Coastal doghobble	LF	NC	Greg Gust	223	93.62	20	RT
84474	*Mahonia bealei*	Mahonia	bealei	Berberidaceae	Leatherleaf mahonia	P	NC	Greg Gust	225	92.47	20	RT
84475	*Pastinaca sativa*	Pastinaca	sativa	Apiaceae	Parsnip	ST	NC	Greg Gust	226	4.28	20	
84476	*Eupatorium fistulosum*	Eupatorium	fistulosum	Asteraceae	Trumpetweed, Joe Pye weed, queen-of-the-meadow	LF-FL	NC	Greg Gust	227	98.69	20	RT
84477	*Clethra acuminata*	Clethra	acuminata	Clethraceae	Mountain sweet pepper bush	LF-FR	NC	Greg Gust	228	65.32	20	
84478	*Clethra acuminata*	Clethra	acuminata	Clethraceae	Mountain sweet pepper bush	ST	NC	Greg Gust	228	61.12	20	
84479	*Pastinaca sativa*	Pastinaca	sativa	Apiaceae	Parsnip	LF-FL	NC	Greg Gust	226	99.82	20	RT
84480	*Pastinaca sativa*	Pastinaca	sativa	Apiaceae	Parsnip	RT	NC	Greg Gust	226	22.57	20	
84481	*Solidago gigantea*	Solidago	gigantea	Asteraceae	Giant Goldenrod	P	NC	Greg Gust	229	17.91	20	
84482	*Halesia carolina*	Halesia	carolina	Styracaceae	Silver-bell tree	LF	NC	Greg Gust	230	0.31	20	
84483	*Laportea canadensis*	Laportea	canadensis	Urticaceae	Canadian wood nettle	P	NC	Greg Gust	231	68.22	20	
84484	*Tilia heterophylla*	Tilia	heterophylla	Malvaceae	White basswood	LF	NC	Greg Gust	232	28.03	20	
84485	*Castanea dentata*	Castanea	dentata	Fagaceae	American sweet chestnut	LF	NC	Greg Gust	233	97.05	20	RT
84486	*Castanea dentata*	Castanea	dentata	Fagaceae	American sweet chestnut	ST	NC	Greg Gust	233	97.64	20	RT
84487	*Viburnum acerifolium*	Viburnum	acerifolium	Adoxaceae	Honeysuckle	PX	NC	Greg Gust	234	10.96	20	
84488	*Rubus odoratus*	Rubus	odoratus	Rosaceae	Thimbleberry	LF-FL	NC	John Stone	3963	99.78	20	RT
84489	*Rubus odoratus*	Rubus	odoratus	Rosaceae	Thimbleberry	ST	NC	John Stone	3963	97.45	20	RT
84490	*Actaea racemosa*	Actaea	racemosa	Ranunculaceae	Black cohosh, black bugbane, black baneberry	LF	NC	John Stone	3964	36.26	20	
84491	*Actaea racemosa*	Actaea	racemosa	Ranunculaceae	Black cohosh, black bugbane, black baneberry	FR	NC	John Stone	3964	99.06	20	RT
84492	*Actaea racemosa*	Actaea	racemosa	Ranunculaceae	Black cohosh, black bugbane, black baneberry	RT	NC	John Stone	3964	62.98	20	
84493	*Vernonia noveboracensis*	Vernonia	noveboracensis	Asteraceae	New York ironweed	LF-FL	NC	John Stone	3965	77.54	20	
84494	*Vernonia noveboracensis*	Vernonia	noveboracensis	Asteraceae	New York ironweed	ST	NC	John Stone	3965	5.46	20	
84495	*Diphylleia cymosa*	Diphylleia	cymosa	Berberidaceae	Umbrella leaf	PX	NC	John Stone	3966	27.52	20	
84496	*Lindera benzoin*	Lindera	benzoin	Lauraceae	Spice bush	PX	NC	John Stone	3967	90.95	20	RT
84497	*Aruncus dioicus*	Aruncus	dioicus	Rosaceae	Goat's beard	PX	NC	John Stone	3968	99.17	20	RT
84498	*Heuchera villosa*	Heuchera	villosa	Saxifragaceae	Hairy alum root	P	NC	John Stone	3969	97.37	20	RT
84499	*Liriodendron tulipifera*	Liriodendron	tulipifera	Magnoliaceae	Tulip tree	LF	NC	John Stone	3970	93.07	20	RT
84500	*Liriodendron tulipifera*	Liriodendron	tulipifera	Magnoliaceae	Tulip tree	ST	NC	John Stone	3970	95.59	20	RT
84501	*Dryopteris intermedia*	Dryopteris	intermedia	Dryopteridaceae	Intermediate wood fern	P	NC	John Stone	3971	95.41	20	RT
84502	*Adiantum pedatum*	Adiantum	pedatum	Pteridaceae	Northern maidenhair	P	NC	John Stone	3972	16.75	20	
84504	*Aralia racemosa*	Aralia	racemosa	Araliaceae	American spikenard	ST	NC	John Stone	3973	14.01	20	
84505	*Fagus grandifolia*	Fagus	grandifolia	Fagaceae	American beech	LF-FR	NC	John Stone	3974	90.47	20	RT
84506	*Fagus grandifolia*	Fagus	grandifolia	Fagaceae	American beech	ST	NC	John Stone	3974	5.74	20	
84507	*Viburnum cassinoides*	Viburnum	cassinoides	Adoxaceae	Withe rod	ST	NC	John Stone	3975	2.21	20	
84508	*Lobelia siphilitica*	Lobelia	siphilitica	Campanulaceae	Great blue lobelia	LF-FL	NC	John Stone	3977	7.87	20	
84509	*Lobelia siphilitica*	Lobelia	siphilitica	Campanulaceae	Great blue lobelia	ST-RT	NC	John Stone	3977	7.55	20	
84510	*Lespedeza bicolor*	Lespedeza	bicolor	Fabaceae	Lespedeza	LF-FL	NC	John Stone	3978	24.15	20	
84511	*Lespedeza bicolor*	Lespedeza	bicolor	Fabaceae	Lespedeza	ST	NC	John Stone	3978	30.10	20	
84512	*Betula lutea*	Betula	lutea	Betulaceae	Yellow birch	LF	NC	John Stone	3979	97.95	20	RT
84513	*Betula lutea*	Betula	lutea	Betulaceae	Yellow birch	ST	NC	John Stone	3979	29.24	20	
84514	*Betula lutea*	Betula	lutea	Betulaceae	Yellow birch	BK	NC	John Stone	3979	5.07	20	
84515	*Hamamelis virginiana*	Hamamelis	virginiana	Hamamelidaceae	Witch hazel	LF	NC	John Stone	3980	96.23	20	RT
84516	*Hamamelis virginiana*	Hamamelis	virginiana	Hamamelidaceae	Witch hazel	ST	NC	John Stone	3980	93.07	20	RT
84517	*Cornus amomum*	Cornus	amomum	Cornaceae	Silky dogwood	PX	NC	John Stone	3981	96.62	20	RT
84518	*Rhododendron maximum*	Rhododendron	maximum	Ericaceae	Rosebay rhododendron	LF-FL	NC	John Stone	3982	92.85	20	RT
84519	*Celastrus orbiculatus*	Celastrus	orbiculatus	Celastraceae	Oriental bittersweet	ST	NC	Greg Gust	236	94.19	20	RT
84520	*Celastrus orbiculatus*	Celastrus	orbiculatus	Celastraceae	Oriental bittersweet	LF	NC	Greg Gust	236	12.57	20	

84521	*Celastrus orbiculatus*	Celastrus	orbiculatus	Celastraceae	Oriental bittersweet	FR	NC	Greg Gust	236	13.97	20	
84522	*Arctium minus*	Arctium	minus	Asteraceae	Lesser burdock	PX	NC	Greg Gust	237	NA	20	
84523	*Osmunda regalis*	Osmunda	regalis	Osmundaceae	Royal fern	PX	NC	Greg Gust	238	0.05	20	
84524	*Osmunda claytoniana*	Osmunda	claytoniana	Osmundaceae	Interrupted fern	PX	NC	Greg Gust	239	1.22	20	
84525	*Lobelia puberula*	Lobelia	puberula	Campanulaceae	Downy lobelia	P	NC	Greg Gust	240	6.83	20	
84526	*Lycopodium tristachyum*	Lycopodium	tristachyum	Lycopodiaceae	Deeproot clubmoss, groundcedar	PX	NC	Greg Gust	241	5.92	20	
84527	*Tamarix ramosissima*	Tamarix	ramosissima	Tamaricaceae	Tamarisk, saltcedar	PX	AZ	Alan Brant	4039	9.25	20	
84528	*Pycnanthemum incanum*	Pycnanthemum	incanum	Lamiaceae	Hoary mountain mint	P	NC	Greg Gust	244	36.24	20	
84529	*Elephantopus carolinianus*	Elephantopus	carolinianus	Asteraceae	Elephantsfoot	P	NC	Greg Gust	246	98.28	20	RT
84530	*Cirsium vulgare*	Cirsium	vulgare	Asteraceae	Common thistle	PX	NC	Greg Gust	247	7.29	20	
84531	*Tradescantia subaspera*	Tradescantia	subaspera	Commelinaceae	Zigzag spiderwort	PX	NC	Greg Gust	248	2.78	20	
84533	*Carya ovata*	Carya	ovata	Juglandaceae	Shagbark hickory	ST	NC	Greg Gust	249	9.98	20	
84535	*Ilex decidua*	Ilex	decidua	Aquifoliaceae	Possumhaw	LF-FR	NC	John Stone	3983	9.64	20	
84536	*Ilex decidua*	Ilex	decidua	Aquifoliaceae	Possumhaw	ST	NC	John Stone	3983	78.98	20	
84537	*Smilax herbacea*	Smilax	herbacea	Smilacaceae	Carrion flower	LF-ST	NC	John Stone	3984	19.96	20	
84538	*Solidago bicolor*	Solidago	bicolor	Asteraceae	White goldenrod, silverrod	P	NC	John Stone	3985	88.41	20	
84539	*Rudbeckia fulgida*	Rudbeckia	fulgida	Asteraceae	Orange coneflower	P	NC	John Stone	3986	5.43	20	
84540	*Aesculus octandra*	Aesculus	octandra	Hippocastanaceae	Yellow buckeye	ST	NC	John Stone	3988	6.74	20	
84541	*Aesculus octandra*	Aesculus	octandra	Hippocastanaceae	Yellow buckeye	FR	NC	John Stone	3988	98.93	20	RT
84542	*Sorbus americana*	Sorbus	americana	Rosaceae	American mountain ash	LF	NC	John Stone	3989	23.45	20	
84543	*Sorbus americana*	Sorbus	americana	Rosaceae	American mountain ash	ST	NC	John Stone	3989	8.44	20	
84544	*Sorbus americana*	Sorbus	americana	Rosaceae	American mountain ash	FR	NC	John Stone	3989	26.01	20	
84545	*Coreopsis pubescens*	Coreopsis	pubescens	Asteraceae	Star tickseed	P	NC	John Stone	3990	19.45	20	
84657	*Phalaris canariensis*	Phalaris	canariensis	Poaceae	Canary grass	P[WHOLE]	Nevada	Elray Nixon	NULL	NA	20	
84658	*Bromus carinatus*	Bromus	carinatus	Poaceae	Californian brome	P[WHOLE]	Nevada	Elray Nixon	NULL	0.80	20	
84659	*Eruca vesicaria*	Eruca	vesicaria	Brassicaceae	Rocketsalad	RT	Nevada	Elray Nixon	NULL	1.10	20	
84660	*Caulanthus lasiophyllus*	Caulanthus	lasiophyllus	Brassicaceae	California mustard	P[WHOLE]	Nevada	Elray Nixon	NULL	2.40	20	
84661	*Coronopus didymus*	Coronopus	didymus	Brassicaceae	Lesser swinecress	P[WHOLE]	Nevada	Elray Nixon	NULL	11.93	20	
84662	*Delphinium andersonii*	Delphinium	andersonii	Ranunculaceae	Anderson's larkspur	P[WHOLE]	Nevada	Elray Nixon	NULL	3.07	20	
84663	*Rumex hymenosepalus*	Rumex	hymenosepalus	Polygonaceae	Canaigre	ST	Nevada	Elray Nixon	NULL	6.15	20	
84664	*Rumex hymenosepalus*	Rumex	hymenosepalus	Polygonaceae	Canaigre	LF	Nevada	Elray Nixon	NULL	20.45	20	
84665	*Centaurea melitensis*	Centaurea	melitensis	Asteraceae	Maltese star thistle	ST-LF-FL	Nevada	Elray Nixon	NULL	8.33	20	
84666	*cf. Ericameria brachylepis*	cf. Ericameria	brachylepis	Asteraceae	Chaparral goldenbush	ST-LF-FL	Nevada	Elray Nixon	NULL	9.42	20	
84667	*Mirabilis biglovii*	Mirabilis	bigelovii	Nyctaginaceae	Bigelow's desert four o'clock, desert wishbone bush	ST-LF-FL	Nevada	Elray Nixon	NULL	97.27	20	RT
84668	*Chamaecrista fasciculata*	Chamaecrista	fasciculata	Fabaceae	Golden cassia, partridge pea	P	FL	Adam Bradley	1209	93.50	20	RT
84669	*Cliftonia monophylla*	Cliftonia	monophylla	Cyrillaceae	Buckwheat tree	LF-FR	FL	John Stone	3427	95.13	20	RT
84670	*Viburnum nudum*	Viburnum	nudum	Adoxaceae	Smooth withe rod, possumhaw	LF-FR	FL	Adam Bradley	1223	8.95	20	
84671	*Viburnum nudum*	Viburnum	nudum	Adoxaceae	Smooth withe rod, possumhaw	LF-ST	FL	Adam Bradley	1226	28.08	20	
84672	*Viburnum cf. nudum*	Viburnum	cf. nudum	Adoxaceae	Smooth withe rod, possumhaw	LF-FR	FL	Adam Bradley	1227	64.21	20	
84673	*Elephantopus elatus*	Elephantopus	elatus	Asteraceae	Tall elephantsfoot	P	FL	Adam Bradley	1232	2.28	20	
84674	*Pinus palutis*	Pinus	palustris	Pinaceae	Pitch pine	BK	FL	Karen Meyer	37	21.85	20	
84675	*Heuchera bracteata*	Heuchera	bracteata	Saxifragaceae	Bracted alumroot	PX	CO	John Stone	3830	95.40	20	RT
84676	*Potentilla fissa*	Potentilla	fissa	Rosaceae	Bigflower cinquefoil	P	CO	John Stone	3829	39.75	20	
84677	*Cornus stolonifera*	Cornus	stolonifera	Cornaceae	Redosier dogwood	LF-FR	CO	John Stone	3834	96.91	20	RT
84678	*Vaccinium erythrocarpum*	Vaccinium	erythrocarpum	Ericaceae	Southern mountain cranberry	PX	NC	John Stone	3991	84.08	20	
84679	*Ceratozamia mexicana*	Ceratozamia	mexicana	Zamiaceae	Mexican Horncone	LF	MO	Greg Gust	1007	8.32	20	
84680	*Philodendron radiatum*	Philodendron	radiatum	Araceae	Philodendron	LF	MO	Greg Gust	967	18.48	20	
84681	*Samanea saman*	Samanea	saman	Fabaceae	Raintree	ST	MO	Andrew Keith Townesmith	665	2.23	20	
84682	*Pinus palutis*	Pinus	palustris	Pinaceae	Pitch pine	LF	FL	Karen Meyer	37	72.20	20	
84683	*Quercus pumila*	Quercus	pumila	Fagaceae	Running oak	LF-ST	FL	Karen Meyer	63	98.72	20	RT
84684	*Sabal palmetto*	Sabal	palmetto	Arecaceae	Cabbage palmetto	LF	FL	Andrew Keith Townesmith	753	9.01	20	
84685	*Pinus serotina*	Pinus	serotina	Pinaceae	Pond pine	LF	FL	Karen Meyer	102	96.61	20	RT
84686	*Lyonia fruticosa*	Lyonia	fruticosa	Ericaceae	Coastal plain staggerbush	ST	FL	Andrew Keith Townesmith	795	93.27	20	RT
84687	*Livistonia chinensis*	Livistona	chinensis	Arecaceae	Chinese Fan Palm	LF	HI	Karen Meyer	321	21.62	20	
84688	*Bactris militaris*	Bactris	militaris	Arecaceae	Bactris palm	LF	HI	Andrew Keith Townesmith	1035	13.98	20	
84689	*Coix lacryma-jobi*	Coix	lacryma-jobi	Poaceae	Job's tears	LF	HI	Andrew Keith Townesmith	1046	12.13	20	
84690	*Rhapis excelsa*	Rhapis	excelsa	Arecaceae	Broadleaf lady palm, bamboo Palm	LF-FR	MO	Andrew Keith Townesmith	344	NA	20	
84691	*Quercus laevis*	Quercus	laevis	Fagaceae	American turkey oak	LF	FL	Andrew Keith Townesmith	800	93.38	20	RT
84692	*Aspidotis densa*	Aspidotis	densa	Pteridaceae	Indian's dream	PX	CA	Andrew Keith Townesmith	885	16.95	20	
84693	*Nageia falcatus*	Podocarpus	macrophyllus	Podocarpaceae	Kusamaki	LF	HI	Karen Meyer	317	33.96	20	

84694	*Aglaonema commutatum*	Aglaonema	commutatum	Araceae	Chinese or Philippine evergreen	LF	HI	Andrew Keith Townesmith	1029	NA	20	
84695	*Oplismenus hirtellus*	Oplismenus	hirtellus	Poaceae	Basketgrass	PX	HI	Karen Meyer	331	NA	20	
84696	*Ceiba pentandra*	cf. Ceiba	pentandra	Bombacaceae	Mallow	FL	HI	Andrew Keith Townesmith	1040	NA	20	
84697	*Zamia furfuracea*	Zamia	furfuracea	Zamiaceae	Cardboard Sago, Jamaican Sago, Mexican cycad	LF	HI	Andrew Keith Townesmith	1042	1.14	20	
84698	*Arenga undaltifolia*	Arenga	undulatifolia	Arecaceae	Aren gelora	LF	HI	Karen Meyer	337	NA	20	
84699	*Desmodium sandwicense*	Desmodium	sandwicense	Fabaceae	Hawai'i ticktrefoil	P	HI	Karen Meyer	345	NA	20	
84700	*Disotis rotundifolia*	Dissotis	rotundifolia	Melastomataceae	Pinklady	P	HI	Karen Meyer	384	NA	20	
84701	*Yucca harrimaniae*	Yucca	harrimaniae	Agavaceae	Spanish bayonet	ST	Utah	Elray Nixon	NULL	NA	20	
84702	*Nicotiana attenuata*	Nicotiana	attenuata	Solanaceae	Coyote tobacco	ST-LF-IFL	Utah	Elray Nixon	NULL	96.98	20	RT
84703	*Vitis arizonica*	Vitis	arizonica	Vitaceae	Canyon grape	stem	Utah	Elray Nixon	NULL	93.70	20	RT
84704	*Caesalpinia pulcherrima*	Caesalpinia	pulcherrima	Fabaceae	Poinciana, peacock flower, red bird of paradise, pride of Barbados	ST	Nevada	Elray Nixon	NULL	96.81	20	RT
84705	*Arundo donax*	Arundo	donax	Poaceae	Giant reed	ST	Nevada	Elray Nixon	NULL	NA	20	
84706	*Atriplex fruticulosa*	Atriplex	fruticulosa	Chenopodiaceae	Ball saltbush	ST-LF-FR	Nevada	Elray Nixon	NULL	NA	20	
84707	*Veronica anagallis-aquatica*	Veronica	anagallis-aquatica	Plantaginaceae	Water speedwell	P[WHOLE]	Utah	Elray Nixon	NULL	1.42	20	
84708	*Agrostis stolonifera*	Agrostis	stolonifera	Poaceae	Creeping bentgrass	P[WHOLE]	Utah	Elray Nixon	NULL	NA	20	
84709	*Ribes montigenum*	Ribes	montigenum	Grossulariaceae	Gooseberry-currant	ST	Utah	Elray Nixon	NULL	93.42	20	RT
84710	*Atriplex hymenelytra*	Atriplex	hymenelytra	Chenopodiaceae	Desert holly	ST	Nevada	Elray Nixon	NULL	NA	20	
84711	*Atriplex lentiformis*	Atriplex	lentiformis	Chenopodiaceae	Quail bush	ST	Nevada	Elray Nixon	NULL	NA	20	
84712	*Quercus alba*	Quercus	alba	Fagaceae	White oak	BK	MO	John Stone	4257	91.28	20	RT
84713	*Encelia farinosa*	Encelia	farinosa	Asteraceae	Brittle bush	RT	CA	Greg Gust	464	69.38	20	
84714	*Hemerocallis fulva*	Hemerocallis	fulva	Liliaceae	Orange Daylily, Tawny Daylily, Tiger Lily, Ditch Lily	LF	MO	Andrew Keith Townesmith	343	NA	20	
84715	*Leea rubra*	Leea	rubra	Vitaceae	West Indian holly, red leea	ST	MO	Andrew Keith Townesmith	364	97.08	20	RT
84716	*Philodendron ferrugineum*	Philodendron	ferrugineum	Araceae	Philodendron	LF	MO	Greg Gust	962	NA	20	
84717	*Urera baccifera*	Urera	baccifera	Urticaceae	Scratchbush	ST	PR	Andrew Keith Townesmith	371	NA	20	
84718	*Ribes missouriense*	Ribes	missouriense	Grossulariaceae	Missouri gooseberry	PX	MO	Tracy Scott	5	NA	20	
84719	*Mahonia aquifolium*	Mahonia	aquifolium	Berberidaceae	Oregon grape	RT	OR	Andrew Keith Townesmith	639	NA	20	
84720	*Coccoloba pubescens*	Coccoloba	pubescens	Polygonaceae	Grandleaf seagrape	ST	MO	Andrew Keith Townesmith	661	94.19	20	RT
84721	*Cercocarpus minutiflorus*	Cercocarpus	minutiflorus	Rosaceae	Smooth mountain mahogany	ST	CA	Karen Meyer	5	NA	20	
84722	*Rhus integrifolia*	Rhus	integrifolia	Anacardiaceae	Lemonade berry	ST	CA	Karen Meyer	8	97.52	20	RT
84723	*Quercus agrifolia*	Quercus	agrifolia	Fagaceae	Encina; coast live oak	FR	CA	Andrew Keith Townesmith	673	NA	20	
84724	*Artemisia californica*	Artemisia	californica	Asteraceae	Wormwood	RT	CA	Andrew Keith Townesmith	676	NA	20	
84725	*Genista monspessulana*	Genista	monspessulana	Fabaceae	French broom	LF-ST	CA	Tracy Scott	118	NA	20	
84726	*Quercus terbinella*	Quercus	turbinella	Fagaceae	Turbinella oak	LF	CA	Karen Meyer	23	48.92	20	
84727	*Adiantum capillis-veneris*	Adiantum	capillus-veneris	Pteridaceae	Maidenhair fern, Avenca	P	CA	Andrew Keith Townesmith	706	NA	20	
84728	*Foeniculum vulgare*	Foeniculum	vulgare	Apiaceae	Fennel	LF	CA	Tracy Scott	155	NA	20	
84729	*Pinus elliotii*	Pinus	elliottii	Pinaceae	Slash pine	ST	FL	Karen Meyer	36	38.09	20	
84730	*Quercus incana*	Quercus	incana	Fagaceae	Bluejack oak	LF	FL	Karen Meyer	47	67.08	20	
84731	*Hypericum hypericoides*	Hypericum	hypericoides	Hypericaceae	St. Andrews cross	ST	FL	Karen Meyer	53	97.91	20	RT
84733	*Asimina obovata*	Asimina	obovata	Annonaceae	Bigflower papaw	LF	FL	Karen Meyer	86	24.04	20	
84734	*Cinnamomum camphora*	Cinnamomum	camphora	Lauraceae	Camphor	LF	FL	Karen Meyer	96	15.25	20	
84735	*Cinnamomum camphora*	Cinnamomum	camphora	Lauraceae	Camphor	BK	FL	Karen Meyer	96	11.94	20	
84736	*Rumex verticillata*	Rumex	verticillatus	Polygonaceae	Swamp dock	ST	FL	Andrew Keith Townesmith	784	19.17	20	
84737	*Nuphar luteum*	Nuphar	luteum	Nymphaeaceae	Water lilly	FL	FL	Karen Meyer	103	NA	20	
84738	*Nuphar luteum*	Nuphar	luteum	Nymphaeaceae	Water lilly	FR	FL	Karen Meyer	103	96.19	20	RT
84739	*Lachnocaulon minus*	Lachnocaulon	minus	Eriocaulaceae	Small's bogbutton	P	FL	Karen Meyer	104	0.37	20	
84740	*Lyonia fruticosa*	Lyonia	fruticosa	Ericaceae	Coastal plain staggerbush	LF-FL	FL	Andrew Keith Townesmith	795	69.79	20	
84741	*Rhus copallinum*	Rhus	copallinum	Anacardiaceae	Shining, winged or dwarf sumac	FR	FL	Andrew Keith Townesmith	771	0.66	20	
84742	*Dombeya tiliacea*	Dombeya	tiliacea	Malvaceae	NULL	ST	MO	Karen Meyer	290	3.52	20	
84743	*Musa ornata*	Musa	ornata	Musaceae	Flowering or ornamental banana	LF	HI	Karen Meyer	306	16.44	20	
84744	*Spathiphyllum wallisii*	Spathiphyllum	wallisii	Araceae	Peace lily	ST	HI	Karen Meyer	304	13.83	20	
131665	Difluoromethylornithine									99	20	

Plant parts BK- stem bark, LF- leaves, FL- flowers, ST- stem, RT-root, FR-fruit ; RT- samples retested in secondary assay

Table S2- Secondary screeneing of active plants extracts against Trypanosoma brucei							
NPID	SampleName	Plant Part	*T. brucei* IC50 (μg/mL)	Standard deviation IC 50	*T brucei* IC90 (mg/mL)	Standard deviation IC 50	THP1 % inhibition at 20 μg/mL
81877	*Ulex europaea*	BK	4.05	0.78	>10	NA	0.00
81880	*Alnus rubra*	BK	0.94	0.58	1.95	0.17	1.98
81883	*Rumex obtusifolius*	FR	3.69	0.06	7.28	0.12	2.61
81886	*Arctostaphylos canescens*	LF	3.27	0.24	6.00	0.07	3.79
81890	*Boykinia major*	RT	2.82	0.44	8.31	1.50	4.69
81892	*Phacelia hastata*	PX	5.03	2.64	7.70	1.42	2.79
81895	*Abies procera*	FR	4.22	0.23	7.03	0.24	6.56
81896	*Abies procera*	BK	3.74	0.22	6.38	0.09	6.93
81897	*Juniperus communis*	LF-ST	2.40	0.44	6.66	2.13	0.00
81899	*Boykinia occidentalis*	P	3.03	0.30	7.04	0.26	0.99
81900	*Gaultheria ovatifolia*	P	3.08	0.07	7.01	0.11	0.78
81907	*Ribes lacustre*	PX	3.62	0.24	6.31	0.41	2.66
81908	*Chrysolepis chrysophylla*	FL	2.89	0.40	7.31	1.57	3.05
81912	*Quercus chrysolepis*	LF	2.62	0.67	>10	NA	3.17
81914	*Hypericum calycinum*	RT	>10	NA	>10	NA	2.94
81918	*Quercus chrysolepis*	BK	3.97	0.29	7.27	0.26	7.21
81919	*Ribes roezlii*	PX	3.21	0.22	6.35	0.13	0.00
81925	*Rhododendron occidentale*	LF	2.87	0.43	6.88	1.16	2.45
81927	*Aralia californica*	FR	5.68	2.39	7.08	1.83	1.86
81929	*Aralia californica*	LF	7.34	0.80	>10	NA	3.44
81932	*Darmera peltata*	LF	3.34	0.14	6.18	0.02	5.71
81940	*Arctostaphylos viscida*	LF	2.88	0.21	5.96	1.09	6.49
81943	*Sanguisorba officinalis*	LF	3.56	0.39	7.50	0.16	7.67
81944	*Sanguisorba officinalis*	ST-FL	3.87	0.22	6.90	0.37	9.86
81946	*Lithocarpus densiflorus*	FR	3.17	0.09	6.18	0.02	0.00
81947	*Pinus lambertiana*	LF	3.18	0.50	6.62	1.28	1.46
81953	*Eriogonum umbellatum*	LF-ST	2.79	0.34	6.25	1.94	0.53
81957	*Arceuthobium campylopodum*	PX	6.88	1.40	>10	NA	4.63
81959	*Ceanothus cordulatus*	PX	4.33	0.18	7.23	0.22	4.29
82402	*Lotus crassifolius*	FR	>10	NA	>10	NA	5.80
82407	*Potentilla gracilis*	ST-FR	3.33	0.33	6.56	0.28	7.36
82411	*Hylocomium cf. splendens*	P	4.78	0.18	7.55	0.17	8.56
82423	*Ludovia lancifolia*	LF	3.73	0.10	5.84	0.18	0.00
82425	*Solandra longifolia*	ST	>10	NA	>10	NA	0.00
82426	*Clerodendron splendens*	LF	>10	NA	>10	NA	1.71
82429	*Quercus ilex*	ST	5.20	0.44	8.37	0.63	2.89
82430	*Medinilla magnifica*	ST	3.96	0.32	6.81	0.32	4.54
82435	*Ceanothus greggi*	LF	3.90	0.18	7.25	0.07	4.62
82436	*Quercus berberidifolia*	LF-ST	3.14	0.32	6.53	0.55	6.06
82438	*Eriogonum fisciculatum*	LF	2.68	0.44	7.73	2.33	9.43
82440	*Rhus integrifolia*	LF	2.97	0.01	4.41	0.29	2.28
82441	*Quercus x acutidens*	ST	>10	NA	>10	NA	1.03
82444	*Baccharis salicifolia*	LF-FL	5.04	0.91	>10	NA	1.20
82446	*Penstemon spectablis*	RT	3.20	0.15	7.02	0.08	3.23
82449	*Alnus rhombifolia*	FL	4.95	0.32	>10	NA	3.25
82451	*Baccharis pilularis*	LF	>10	NA	>10	NA	1.54
82453	*Hoita macrostachya*	LF	0.48	0.00	0.62	0.00	3.32
82460	*Ceanothus spinosa*	LF	3.38	0.22	7.99	0.05	6.11
82461	*Ceanothus spinosus*	ST	4.19	0.11	7.55	0.20	3.24
82463	*Mimulus longiflorus*	RT	4.08	0.51	7.89	1.33	3.31
82466	*Lepechinia calycina*	LF	2.50	0.05	5.04	0.12	3.91
82467	*Ribes speciosum*	LF-ST-FL	2.95	0.10	5.90	0.08	3.70
82468	*Salvia spathacea*	ST	1.13	0.78	3.46	0.34	6.86
82484	*Sabal minor*	FL	1.06	0.44	2.07	0.96	4.93
83280	*Quercus geminata*	LF	7.72	0.23	>10	NA	5.16
83284	*Pinus serotina*	BK	8.37	0.51	9.86	0.12	7.08
83289	*Eriocaulon anceps*	P	4.89	3.51	>10	NA	6.05

83291	*Salix caroliniana*	LF	2.24	1.52	>10	NA	8.02
83294	*Berlandiera subacaulis*	LF	>10	NA	>10	NA	7.21
83307	*Eriogonum nudum*	ST-FL	6.22	2.92	8.39	1.43	8.32
83309	*Arceuthobium occidentale*	P	2.47	2.23	>10	NA	9.71
83317	*Spathiphyllum wallisii*	LF	2.78	NA	>10	NA	8.93
83319	*Syzygium malaccense*	LF	2.76	0.46	>10	NA	8.35
83323	*Elaeocarpus angustifolius*	LF	3.22	0.29	8.47	0.55	10.31
83329	*Syzygium aqueum*	LF	1.84	NA	>10	NA	6.32
83334	*Medinilla magnifica*	FL-FR	2.25	1.16	7.89	2.48	4.30
83335	*Ficus trigonata*	LF	>10	NA	>10	NA	8.92
83345	*Eucalyptus citriodora*	LF	2.91	0.21	5.82	0.04	4.30
83347	*Schefflera actinophylla*	LF	4.59	0.80	>10	NA	4.79
83351	*Eucalyptus citriodora*	BK	2.63	0.78	>10	NA	6.09
83357	*Ardisia elliptica*	LF	>10	NA	>10	NA	7.37
83358	*Nymphaea odorata*	FL	3.53	0.06	6.33	0.02	9.26
83360	*Acer rubrum*	LF	2.88	0.50	7.45	0.70	6.29
83361	*Acer rubrum*	ST	2.06	1.63	>10	NA	7.67
83364	*Verbesina virginica*	LF-FL	3.19	0.24	6.61	0.18	4.75
83938	*Chamaecrista nictitans*	P	5.76	0.73	>10	NA	7.86
83944	*Geranium richardsonii*	P	3.26	0.05	6.86	0.57	8.29
83947	*Arnica cordifolia*	P	>10	NA	>10	NA	8.30
83954	*Pinus aristata*	P	2.42	0.17	>10	NA	8.08
83956	*Vaccinium scoparium*	P	>10	NA	>10	NA	8.41
83957	*Arnica mollis*	P	>10	NA	>10	NA	0.00
83959	*Saxifraga odontoloma*	P	>10	NA	>10	NA	0.00
83961	*Ribes montigenum*	PX	2.98	0.91	>10	NA	0.94
83963	*Rubus deliciosus*	PX	3.99	0.20	7.04	0.16	2.27
83972	*Ribes cf. leptanthum*	PX	7.38	0.98	>10	NA	5.87
83981	*Convolvulus arvensis*	P	>10	NA	>10	NA	6.22
83986	*Yucca glauca*	ST	3.56	0.49	>10	NA	8.34
83991	*Bahia oppositifolia*	P	3.77	1.01	5.51	1.15	8.46
83995	*Senecio spartioides*	PX	>10	NA	>10	NA	0.00
84003	*Magnolia fraseri*	ST	7.29	0.32	>10	NA	0.00
84006	*Asclepias exaltata*	FR	4.13	3.85	>10	NA	0.98
84008	*Rhododendron catawbiense*	LF	4.17	1.37	>10	NA	1.99
84009	*Rhododendon catawbiense*	ST	>10	NA	>10	NA	4.42
84010	*Rhododendron catawbiense*	FR	4.45	0.00	7.86	0.02	5.39
84014	*Ilex ambigua*	ST	3.41	0.15	6.46	0.21	4.84
84020	*Elaeagnus umbellata*	ST	>10	NA	>10	NA	10.20
84022	*Tsuga canadensis*	PX	4.07	0.21	7.04	0.16	0.00
84023	*Acer pensylvanicum*	ST	3.20	0.20	6.14	0.18	0.17
84024	*Helianthus giganteus*	PX	3.03	0.09	6.05	0.02	1.11
84463	*Viburnum nudum*	FR	>10	NA	>10	NA	2.61
84465	*Diervilla sessilifolia*	ST	3.76	0.80	6.38	0.65	3.30
84466	*Vaccinium pallidum*	PX	>10	NA	>10	NA	4.55
84467	*Magnolia tripetala*	LF	3.06	0.18	4.96	0.45	4.36
84468	*Magnolia tripetala*	FR	5.97	2.41	>10	NA	6.74
84470	*Ligustrum sinense*	LF-FR	2.77	0.40	4.41	1.46	0.00
84471	*Ligustrum sinense*	ST	4.90	2.74	7.19	1.85	0.00
84472	*Leucothoe axillaris*	ST	7.54	0.17	9.31	0.02	0.00
84473	*Leucothoe axillaris*	LF	7.63	0.36	9.31	0.12	0.00
84474	*Mahonia bealei*	P	9.18	NA	>10	NA	3.46
84476	*Eupatorium fistulosum*	LF-FL	>10	NA	>10	NA	4.13
84479	*Pastinaca sativa*	LF-FL	8.50	1.11	>10	NA	4.87
84485	*Castanea dentata*	LF	>10	NA	>10	NA	7.03
84486	*Castanea dentata*	ST	4.57	NA	8.34	1.67	0.00
84488	*Rubus odoratus*	LF-FL	1.95	2.01	>10	NA	0.00
84489	*Rubus odoratus*	ST	5.58	1.05	>10	NA	0.00
84491	*Actaea racemosa*	FR	3.40	0.28	5.20	0.16	2.17
84496	*Lindera benzoin*	PX	4.94	NA	>10	NA	3.99
84497	*Aruncus dioicus*	PX	5.74	5.53	>10	NA	5.94

84498	*Heuchera villosa*	P	3.50	1.12	5.96	0.70	7.42
84499	*Liriodendron tulipifera*	LF	4.75	0.39	7.82	0.10	9.84
84500	*Liriodendron tulipifera*	ST	>10	NA	>10	NA	0.00
84501	*Dryopteris intermedia*	P	4.29	3.61	>10	NA	0.00
84505	*Fagus grandifolia*	LF-FR	6.47	2.20	>10	NA	1.59
84512	*Betula lutea*	LF	6.26	0.99	>10	NA	2.31
84515	*Hamamelis virginiana*	LF	3.29	0.24	5.75	0.16	2.53
84516	*Hamamelis virginiana*	ST	2.54	0.53	4.65	3.92	2.89
84517	*Cornus amomum*	PX	3.24	0.61	8.07	1.19	3.53
84518	*Rhododendron maximum*	LF-FL	4.24	0.13	8.24	0.08	5.61
84519	*Celastrus orbiculatus*	ST	>10	NA	>10	NA	0.00
84529	*Elephantopus carolinianus*	P	4.76	NA	>10	NA	0.00
84541	*Aesculus octandra*	FR	5.74	5.53	>10	NA	37.87
84667	*Mirabilis biglovii*	ST-LF-FL	3.50	1.12	5.96	0.70	0.00
84668	*Chamaecrista fasciculata*	P	4.75	0.39	7.82	0.10	1.58
84669	*Cliftonia monophylla*	LF-FR	>10	NA	>10	NA	2.52
84675	*Heuchera bracteata*	PX	4.29	3.61	>10	NA	4.66
84677	*Cornus stolonifera*	LF-FR	6.47	2.20	>10	NA	7.68
84683	*Quercus pumila*	LF-ST	6.26	0.99	>10	NA	0.00
84685	*Pinus serotina*	LF	3.29	0.24	5.75	0.16	0.00
84686	*Lyonia fruticosa*	ST	2.54	0.71	4.65	3.92	0.00
84691	*Quercus laevis*	LF	3.24	0.61	8.07	1.19	1.89
84702	*Nicotiana attenuata*	ST-LF-IFL	4.24	0.13	8.24	0.08	1.65
84703	*Vitis arizonica*	stem	>10	NA	>10	NA	1.83
84704	*Caesalpinia pulcherrima*	ST	4.76	NA	>10	NA	2.17
84709	*Ribes montigenum*	ST	1.94	0.67	7.39	2.74	6.82
84712	*Quercus alba*	BK	1.42	0.30	7.53	0.44	0.00
84715	*Leea rubra*	ST	1.62	0.32	4.50	1.04	0.00
84720	*Coccoloba pubescens*	ST	0.83	0.04	1.91	0.16	0.00
84722	*Rhus integrifolia*	ST	0.50	0.10	1.07	0.33	0.00
84731	*Hypericum hypericoides*	ST	2.28	1.03	>10	NA	0.00
84738	*Nuphar luteum*	FR	0.42	0.02	1.32	0.11	0.00
131665	Difluoromethylornithine		5.07	0.27	12.37	0.96	1.40
103650	Pentamidine		0.002	0.001	0.003	0.001	0.00
**Plant parts BK- stem bark, LF- leaves, FL- flowers, ST- stem, RT-root, FR-fruit**							
